# Metalo components exhibiting significant anticancer and antibacterial properties: a novel sandwich-type like polymeric structure

**DOI:** 10.1038/s41598-020-69416-x

**Published:** 2020-07-27

**Authors:** Ahmet Karadağ, Nesrin Korkmaz, Ali Aydın, Hüseyin Akbaş, Şaban Tekin, Yusuf Yerli, Fatih Şen

**Affiliations:** 1 Department of Chemistry, Faculty of Arts and Sciences, Yozgat Bozok University, 66200 Yozgat, Turkey; 2 Department of Biotechnology, Faculty of Science, Bartın University, 74100 Bartın, Turkey; 3 Department of Basic Medical Science, Faculty of Medicine, Bozok University, 66200 Yozgat, Turkey; 4 Department of Chemistry, Faculty of Art and Science, Tokat Gaziosmanpaşa University, 60250 Tokat, Turkey; 5 TÜBİTAK MRC Genetic Engineering and Biotechnology Institute, 41470 Gebze, Turkey; 6 Department of Basic Medical Sciences, Medical Biology, Faculty of Medicine, University of Health Sciences, 34668 Istanbul, Turkey; 7 Physics Department, Art and Science Faculty, Yıldız Technical University, 34220 Istanbul, Turkey; 8 Biochemistry Department, Sen Research Group, Faculty of Arts and Science, Dumlupınar University, Evliya Çelebi Campus, 43100 Kütahya, Turkey

**Keywords:** Biochemistry, Biotechnology, Cancer, Microbiology, Chemistry, Materials science

## Abstract

Four new dicyanoargentate(I)-based complexes **1**–**4** were synthesized from certain metal ions with a tetradentate ligand [N, N-bis (2-hydroxyethyl) -ethylenediamine; *N-bishydeten*] and determined by diverse procedures (elemental, thermal, FT-IR, ESI–MS for **1**–**3** and, magnetic susceptibility and EPR for **1**, and **2**) including crystal analysis of **4**. The crystal method revealed that complex **4** has a sandwich-type like polymeric chemical structure with layers formed by [Cd(*N-bishydeten*)_2_]^2+^ cations and [Ag(CN)_2_]^−^ anions. The complexes were further characterized by fluorescence and UV spectroscopy to determine their physicochemical features. The complexes displayed a DNA binding activity within the same range as found for cisplatin, in addition to their strong stability in the presence of the physiological buffer system. The complexes were also investigated for pharmacological properties like interaction with DNA/Bovine serum albumin, anticancer and antibacterial activities. Physicochemical studies of DNA with the complexes suggested that the interaction mode between them are possibly both intercalative and groove binding types. These spectroscopic measurements also show that there may be a binding tendency between BSA and the complexes via hydrogen or Van der Waals bonds. The viability tests demonstrated that all the complexes exhibited antibacterial (**1**–**4**) and anticancer effects (**2**–**4**) toward ten diverse bacterial strains and three tumor cells (HT-29 colon adenocarcinoma, HeLa cervical cancer, and C6 glioma), respectively.

## Introduction

Despite the current advances in cancer therapy, the death rate from cancer and therapeutic agents are still increasing^1^. A significant number of studies have focused on the synthesis and design of a new antiproliferative agent to reduce the risk of drug resistance and cell toxicity^2–4^. Some of these studies are related to metallo compounds which constitute a significant part of agents with potent pharmacological activities, and their medicinal availability has been still explored^5,6^. For example, some macrocyclic polyamines containing Ni, Cu, and Ru could recognize TAR RNA molecules and cleave them, and affect the interaction of Tat-RNA^7^. Mn(II) complex of 2H-5-hydroxy-1,2,5-oxadiazo[3,4-f]1,10-phenanthroline has significant antitumor activity against HL-60, KB, Hela and BGC-823 cells. This compound binds with DNA by intercalating via the ligand L^8^. Mn(II) complex of 6,7-dicycanodipyridoquinoxaline intercalates into DNA base pairs via the ligand L and has significant antitumor properties towards HL-60, KB, Hela and BGC-823 cells. This compound exhibit high antiproliferative effects within a μM range similar to those of antitumor drug 5-fluorouracil (5-FU)^9^. An another example of these metallo compounds with potent pharmacological activities is currently shown by cisplatin molecule and its analogs like carboplatin or oxaliplatin compounds, which have most widely prescribed metal-based anticancer drugs to treat a variety of cancer cases like lymphomas, lung, bladder, ovarian, and germ cell tumors^10^. 5-FU and cisplatin derivatives are still widely used anticancer drugs in the treatment of colon, breast and other cancers. Due to their unique chemical structure, they can easily bind to DNA and therefore interferes with nucleoside metabolism leading to growth inhibition^11,12^. In subsequent studies, many metallo compounds against cancer were synthesized, characterized and tested to determine their pharmacological properties^13–15^. However, scientists are still in need for novel approaches to tackle the limitations of cancer treatment. Therefore, many research groups are trying to investigate new metallo-compounds with high efficacy and low toxicity, as an alternative to cisplatin. In this context, many silver or other metallo compounds with promising antitumor activity have been introduced^16–27^. Among these, cyanido complexes are one of the alternatives that can be used in cancer therapy. Our studies showed that these complexes exhibit excellent anticancer, antibacterial and even antifungal activity^28–34^.

Cyanido metal complexes with *d*^10^ metal centers Ag(I), and Au(I) ions are two-coordinated structures in [Ag(CN)_2_]^−^ and [Au(CN)_2_]^−^ anionic forms having linear geometries. Both building blocks are ideal units to discover the utilize of argentophilicity or aurophilicity as a supramolecular design element of coordination polymer synthesis^35–38^. Among the cyanidometallates, Ag(I) polymers have high labiality of the Ag–donor bond and therefore, the structures of Ag(I) coordination polymers, which are usually crystallizable, can be often determined by X-ray crystallography^39,40^. On the other hand, such coordination polymers attract considerable attention owing to their widespread usage or applications^37,41,42^.

In this work, our group reported synthesis, structural characterization and some properties of polymeric [Ni(*N*-*bishydeten*)Ag_3_(CN)_5_] (**1**), [Cu(*N-bishydeten*)Ag_3_(CN)_5_] (**2**), [Zn(*N-bishydeten*)Ag_3_(CN)_5_] (**3**) and [Cd(*N-bishydeten*)]_4_[Ag(CN)_2_]_8_[Ag(CN)]_2_ (4) along with the results of the X-ray structure assay of complex **4**. Here, we focused on the analysis of the complex-DNA/BSA interaction together with their pharmacological activities like antibacterial (**1**–**4**), anticancer and cytotoxic properties (**2**–**4**) by using various powerful methods such as Lactate Dehydrogenase (LDH) Cytotoxicity^43^ and BrdU Cell ELISA assays^44^. The action mechanisms of **2**–**4** were also explored by using DNA laddering^45^, TUNEL, Topoisomerase I inhibitor activity, and cell migration assays. The results showed that complexes **2**–**4** were highly antiproliferative with low cytotoxic, and apoptotic characteristics. Furthermore, they suppressed Topoisomerase I activity and cell migration. Accordingly, we suggest that the complexes have a potential for use as novel anticancer drugs.

## Experimental section

### Synthesis

**Figure 1:**
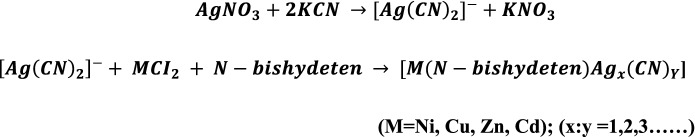
General reaction scheme of complex.

The synthesis acts were performed in the room temperature. The KCN (153 mg, 1.175 mmol) was added into a magnetically stirred solution of AgNO_3_ (200 mg, 1.177 mmol) in ethyl alcohol (20 mL)/water (10 mL). Firstly, the Ni(II), Cu(II), Zn(II) and Cd(II) salts (1 mmol) were added to the clear solution of K[Ag(CN)_2_] (1 mmol, 0.199 g) prepared in the water–ethyl alcohol (volume ratio of 2:1) mixture. Afterward, the obtained metal salt solution was added to the auxiliary ligand *N-bishydeten* (2 mmol, 0.296 g) solution prepared in the alcohol, and it was stirred for about one hour. The resulting product was filtered, and also the clear filtrate was left to crystallize under room conditions. Complexes **1**–**3** were obtained in low yields as powder crystals, while complex **4** also formed in low yields, but as single crystals (Table S1), (Fig. 1). The reason that complexes are obtained in low yields may be a consequence of the very high tendency of *N-bishydeten* to produce stable complexes in the solution media or due to the steric hindrance around the coordination centre^30,33,46–51^.

#### [Ni (***N-bishydeten***)Ag_3_(CN)_5_] (1)

Pink precipitates were recorded with a yield of 43% for **1**. Anal. Calc. for C_11_H_16_N_7_O_2_Ag_3_Ni (%) C, 20.00; H, 2.44; N, 14.84 Found (%): C, 19.68; H, 2.14; N, 14.78. IR (KBr disk; cm^−1^) 3596 υ_OH;_ 3336, 3280, 3119 υ_NH_; 2979, 2904, 2861 υ_CH_; 2163, 2129 υ_C≡N_; 1452 δ_N–H_; 1197 υ_CN_; 1031 υ_CO_. The effective magnetic moment, *μ*_eff_ (Bohr magnetons, *μ*_B_); *μ*_*eff*E_ (*μ*_*eff*T_) values (*μ*_S+L_; Magnetic moments with spin-orbital contributions) for **1** (Ni^2+^, *d*^*8*^): 4.52 (4.47)^52^. ESI-HR (m/z) [100%; M + 2H]^+^ 658.49; analysis for **1** (656.78).

#### [Cu(***N-bisydeten***)Ag_3_(CN)_5_] (2)

Light green precipitates were recorded with 40% yield for **2**. Anal. Calc. for C_11_H_16_N_7_O_2_Ag_3_Cu (%): C, 19.85; H, 2.42; N, 14.73 Found (%): C, 20.41; H, 2.91; N, 12.22. IR (KBr disk; cm^−1^) 3081 υ_OH_; 3313, 3235 υ_NH_; 2915, 2869, 2846 υ_CH_; 2134, 2125 υ_C≡N_; 1473, 1450 δ_N–H_; 1141 υ_CN_; 1062 υ_CO_. The effective magnetic moment, *μ*_eff_ (Bohr magnetons, *μ*_B_); *μ*_*eff*E_ (*μ*_*eff*T_) values (*μ*_S+L_; Magnetic moments with spin-orbital contributions) for **2** (Cu^2+^, *d*^*9*^): 2.17 (3.00)^53^. ESI-HR (m/z) [100%; 2H + M]^+^ 663.37;analysis for **2** (661.78).

#### [Zn(***N-bishydeten***)Ag_3_(CN)_5_] (3)

Colorless precipitates were recorded with a 36% yield for **3**. Anal. Calc. for C_11_H_16_N_7_O_2_Ag_3_Zn (%): C, 19.80; H, 2.42; N, 14.69 Found (%): C, 20.68; H, 2.70; N, 13.60. IR (KBr disk;cm^-1^) 3140 υ_OH_; 3239, 3141 υ_NH_; 2971, 2954, 2883, 2840 υ_CH_; 2161, 2119 υ_C≡N_; 1473, 1455 δ_N–H_; 1122, 1101 υ_CN_; 1064, 1018 υ_CO_. HR-ESI (m/z) [100%; M + 2H]^+^ 664.46; analysis for **3** (662.78).

#### [Cd(***N-bishydeten***)]_4_[Ag (CN)_2_]_8_[Ag (CN)]_2_ (4)

Crystals for this molecule were investigated with 38% yield for **4**. Anal. Calc. for C_42_H_64_Ag_10_Cd_4_N_26_O_8_ (%): C, 19.48; H, 2,49; N, 14.06 Found (%): C, 20.37; H, 2.78; N, 14.26. IR (KBr disk; cm^−1^) 3342–3282 υ_OH_ and υ_NH_; 2964, 2894, 2848 υ_CH_; 2156 υ_C≡N_; 1467, 1446 δ_N–H_; 1114 υ_CN_; 1079, 993 υ_CO_.

### Characterization of 1–4

The structures of complexes **1**–**4** were determined by elemental analysis, IR, EPR (for **1** and **2**), ESI–MS (for **1**–**3**) and X-ray crystallography (for **4**) techniques, and the proposed molecular formulas were estimated by thermal analyses (DTA and TG/DTG) and magnetization measurement (for **1** and **2**) techniques. Thermal analysis is like fingerprinting of materials, such that, each obtained thermal analysis curve is specific to the tested specimen, provided that a correct structure is proposed. For instance, the mass loss indicated by the TG curve of a synthesized compound can be interpreted correctly only if a correct molecular formula is introduced. Besides, the experimental effective magnetic moment (*μ*_*eff*E_) of a complex is consistent with the theoretical effective magnetic moment (*μ*_*eff*T_) to the extent that a correct molecular formula makes the calculation. On the other hand, the typical peaks appearing in the ESI–MS spectra of **1**–**3** are attributed to [M + 2H]^+^. According to the characterization results, complexes **1**–**3** may have a molecular structure as given in Figure S1.

**Figure 2:**
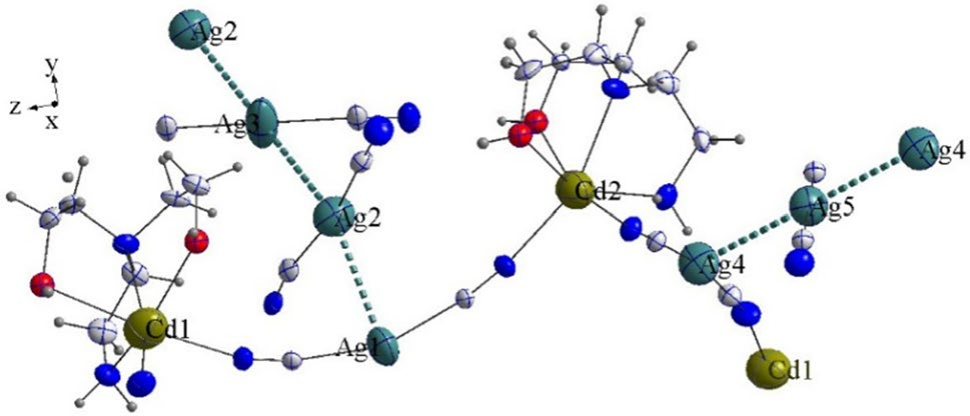
Asymmetric unit structure with argentophilic interactions of complex **4**. (The image in this figure was generated by using K. Brandenburg, Diamond-Crystal and Molecular Structure Visualization, Crystal Impact GbR, Vers. 4.5.2, Bonn, Germany, 2018.)

**Figure 3:**
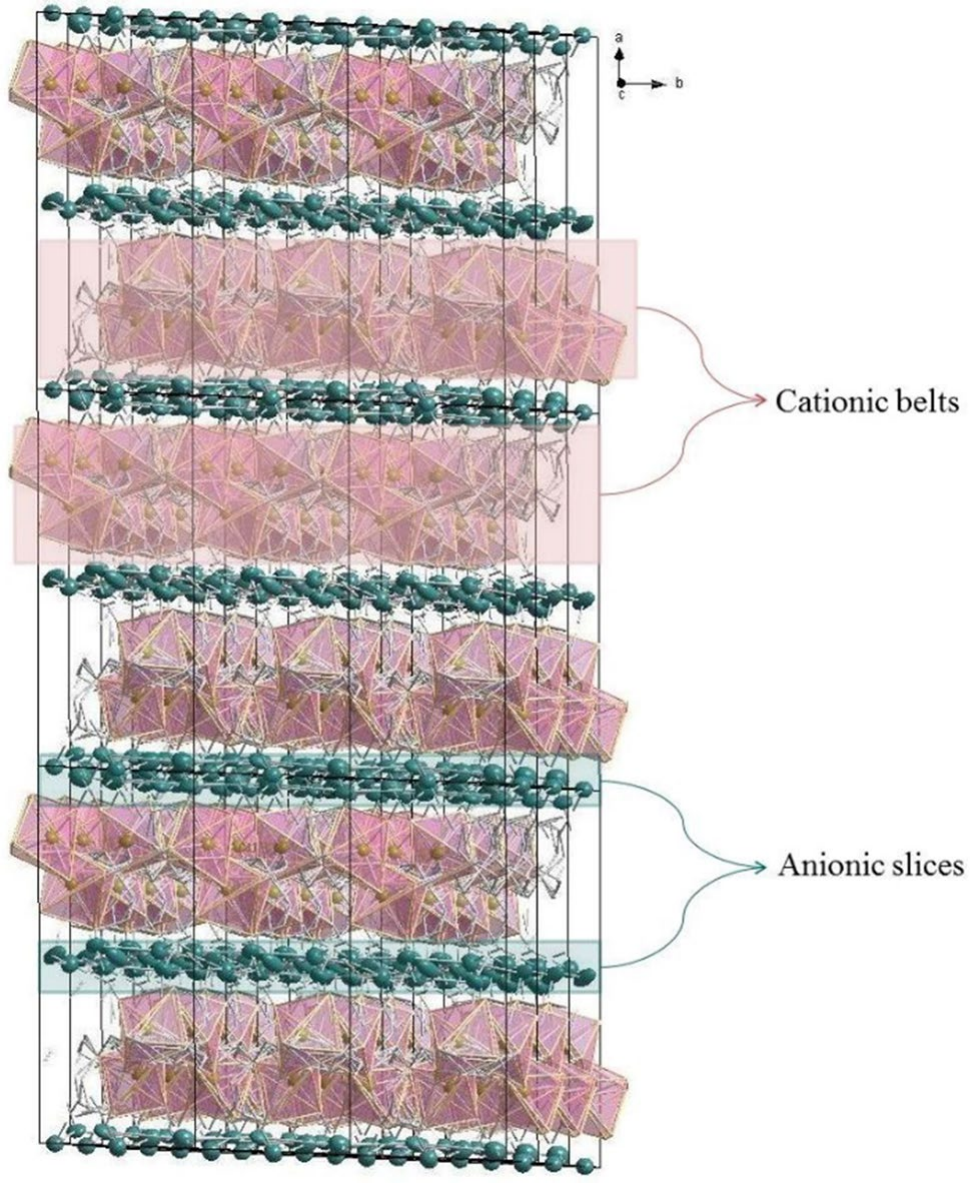
The sandwich-type like the structure of complex **4**. H atoms were omitted, and C, N and O atoms were made invisible. (The image in this figure was generated by using K. Brandenburg, Diamond-Crystal and Molecular Structure Visualization, Crystal Impact GbR, Vers. 4.5.2, Bonn, Germany, 2018.)

The images in Figs. 2 and 3, s5, s6, s7, and s8 were generated by using K. Brandenburg, Diamond-Crystal and Molecular Structure Visualization, Crystal Impact GbR, Vers. 4.5.2, Bonn, Germany, 2018.

## Results and discussion

### IR spectra

The characteristic bands of the functional groups of all the complexes are presented in the "Experimental Section" and the IR spectra of the complexes and *N-bishydeten* ligand are depicted in Figure S2 (Supplementary Material). The most significant vibration frequency for cyanido complexes is known to be the peaks of the CN group. The frequency of the free CN group is different from that of the CN group, which is coordinated with the metal (M–C≡N) or bridged between metal centers (M–C≡N–M′). When the cyano group forms a bridge between the metal centers, the stretching vibration band of cyano usually splits as well, while the vibration frequency value shifts to a higher wavelength^54^. As clearly indicated by the IR spectra of **1**–**4** given in Figure S2, CN stretching vibration peaks have both shifted to higher frequencies, and the peaks are split. The splitting of cyano-stretching vibration peaks in **1**–**3** into two or three peaks is the most critical evidence that they are in the polymeric structures. Meanwhile, the polymeric complex **4** formed a single stretching vibration band (Figure S2). In this part, the terminal cyanido ligand involved in hydrogen bonding (HBs) interactions (Table 1)^55,56^.

**Table 1:** Hydrogen bonds (Å, o) for 4.

	d(D–H)	d(H⋯A)	d(D⋯A)	< (DHA)	Symmetry codes
N(2A)–H(2A) ⋯N(6)	0.90	2.62	3.407	145.87	− *x* + 1, − *y* + 1, − z + 1
N(2A)–H(2A) ⋯N(14)	0.90	2.15	2.994	155.44	− *x* + 1, − *y* + 1, − z + 1
O(1A)–H(1A) ⋯N(14)	0.82	2.52	3.130	132.30	*x* + 1, − *y* + 3/2, z + 1/2
O(2A)–H(2A) ⋯N(12)	0.82	2.07	2.880	168.63	− *x* + 1, *y* + 1/2, − z + 1/2
N(2B)–H(2B) ⋯N(14)	0.90	2.09	2.970	164.05	− *x* + 1, − *y* + 1, − z + 1
O(1B)–H(1B) ⋯N(14)	0.82	2.37	2.996	132.96	*x* + 1,− y + 3/2, z + 1/2
O(2B)–H(2B) ⋯N(12)	0.82	2.05	2.820	155.79	− *x* + 1, *y* + 1/2, − z + 1/2
O(3A)–H(3A) ⋯⋯N(6)	0.82	2.00	2.773	156.27	–
O(4A)–H(4A) ⋯N(5)	0.82	2.16	2.977	171.23	–
N(9A)–H(9A) ⋯N(5)	0.90	2.44	3.243	148.69	− *x* + 1, *y* − 1/2, − z + 1/2
N(9A)–H(9A) ⋯N(12)	0.90	2.60	3.340	140.07	–
O(3B)–H(3B) ⋯N(6)	0.82	1.87	2.650	156.36	–
O(4B)–H(4B) ⋯N(5)	0.82	2.27	2.049	157.70	–
N(9B)–H(9B) ⋯N(5)	0.90	2.13	3.021	170.44	− *x* + 1, *y* − 1/2, − z + 1/2
N(9B)–H(9B) ⋯N(12)	0.90	2.57	3.369	148.07	–

The sharp shift of υ(O–H) over 3000 cm^−1^ to a frequency higher than the O–H vibration of the ligand results from the free presence of the O–H group in the complexes. The υ(NH_2_) stretching vibration of the *N-bishydeten* ligand, which is expected to emerge as a splitting peak from NH_2_-groups, also appeared at 3300–3100 cm^−1^. Excessive splitting of the υ(C–H) stretching vibrations at 2980–2840 cm^−1^ may result from the different environment of the CH_2_ groups as a result of the binding of the *N-bishydeten* ligand to the metal ions.

In addition, the absorption bands observed in the complexes in the range of 1600–993 cm^−1^ are stretching and bending vibrations caused by bonds between the atoms of C–, N–, O– and H–. These absorption bands are also present in the free ligand, N-*bishydeten*, but minor variations in the absorption bands of these functional groups have been observed in the complexes. For example, while the C–N stretching vibration was observed at 1151 cm^−1^ in the neutral ligand, this stretching vibration band in the complexes **1**–**4** was observed at 1197, 1141, 1122 and 1114 cm^−1^, respectively. Also, as a result of the coordination of Ag^I^ and M^II^ ions with C–, O– and N– atoms, peaks which are thought to belong to Ag^I^–C, M^II^–N and M^II^–O stretching vibrations in the range of 600–400 cm^−1^ were found.

On the other hand, the characteristic vibration frequency bands of the neutral ligand *N-bishydeten* in **1**–**4** complexes can be seen as another important evidence of the formation of the expected structures (Figure S2).

### Thermal analyses

Thermogravimetric/thermogravimetric derivative–differential thermal analysis (TG/DTG–DTA) measurements of **1**–**4** also support the crystal composition (4) and the proposed structures (**1**–**3**) as shown in Figures S3 and S4. The TG/DTG curves of **1**–**4** are followed by a process in which a multi-step weight loss is observed from 35 to 1050 °C. The sharp peak at 300–400 °C in the thermal decomposition graph of complex **1** corresponds to an *N-bishydeten* ligand and two cyanide groups, while complex **2** corresponds only to the degradation of the *N-bishydeten* ligand at 500 °C (Figure S4). The neutral *N-bishydeten* ligand is degraded in the initial steps of the thermal decomposition which is followed by the thermal degradation of the cyanido ligand. Finally, the final stage of the thermal decomposition is the temperature at which the inorganic components corresponding to the metal residues are located. Experimental data indicate that the mass remaining in the thermal degradation for complexes **1**–**4** at 1050 °C is the weight corresponding to the inorganic components consisting of Ni + 3Ag (calc.:57.87; found:58.82), Cu + 3Ag (calc.:58.18; found:57.46), Zn + 3Ag (calc.:58.29; found:58.95) and Cd + 10Ag (calc.:45.99; found:45.77), respectively.

### The crystal structure of 4

X-ray analysis revealed that **4** consists of an asymmetric unit, –CN–Cd1(*N-bishydeten*)–NC–Ag1–CN–Cd2(*N-bishydeten*)–NC–Ag4–CN–Cd1(*N-bishydeten*)–NC–, at a three-dimensional zigzag chain structure similar to the "M" shape (Fig. 2 and Figure S5; Supplementary Material). In the polymer chains like the structure of complex **4**, the dicyano silver moieties were adopted slightly bent like conformer using intramolecular argentophilic interaction (Ag1…Ag2…A3 and Ag4…Ag5…Ag4) (Fig. 2 and Figure S6; Supplementary Material). In the structure, argentophilic interactions cooperatively act with HBs interaction (Table 1) which results in a stable macromolecular clustered structure.

**Scheme 1:**
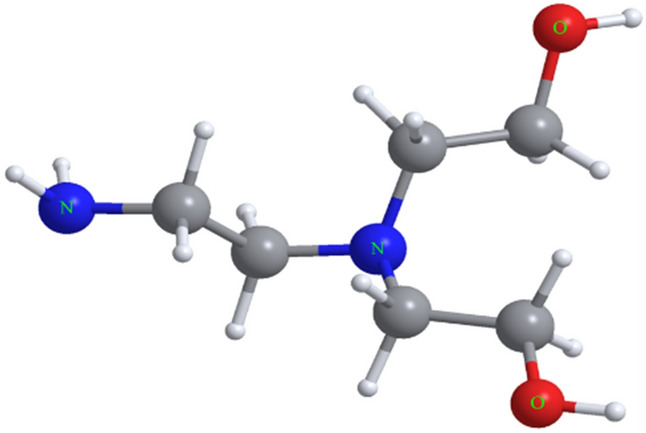
The molecular structure of *N-bishydeten* (ChemDraw Ultra 12.0).

The macromolecular structure is composed of a mixture of Cd/Ag sandwich-type like Cd–N, Cd–O and Ag–N clusters, in which the six coordinated {[Cd(*N-bishydeten*)(*μ*–NC–)]_4_}^8+^ cationic belts are sandwiched between anionic slices {[Ag(*μ*–CN–)_2_]_8_[Ag(CN)]_3_}^8−^ (Fig. 3 and Figures S7, S8; Supplementary Material). The centroid to centroid distance between each repeating cationic and anionic junction is 5.001 Å (Figure S7; Supplementary Material). The six-coordinated {[Cd(*N-bishydeten*)(*μ*–NC–)]_4_}^8+^ cationic belts contain Cd1 and Cd2 metal centers which are located in different planes and surrounded by the *2O-* and 2*N-*atoms of tetradentate *N-bishydeten* ligand and the 4*N-*atoms of bridged dicyanoargentate anions (Ag1 and Ag4) (Fig. 2). The four Cd–O and four Cd–N distances for coordinated two *N-bishydeten* ligands to the Cd1 and Cd2 centers (Table S2; Supplementary Material) are 2.451(11) (Cd1–O1A), 2.392(11) (Cd1–O2B) and 2.429(18) (Cd2–O3A), 2.559(16) Å (Cd2–O4B) and 2.341(4) (Cd1–N1), 2.296(11) (Cd1–N2B) and 2.356(4) (Cd2–N8), 2.326(10) Å (Cd2–N9A), while the Cd–N distances for coordinated four *μ-*ciyanido nitrogens to the Cd1 and Cd2 centers are 2.192(4) (Cd1–N13), 2.311(4) (Cd1–N4) and 2.229(4) (Cd2–N7), 2.302(4)Å (Cd2–N11). The significant differences between the Cd–O and Cd–N bond lengths can be attributed to the zigzag chain structure of M-shape formed in different planes (Figure S5). On the other hand, All the N–Cd–N, C–N–Cd and C–Ag–C which deviates remarkably from the 90° and 180°, which were likely the outcome of the steric limitations arising from the form of the ligands (Scheme 1). The N2A–Cd1–N1, O2A–Cd1–O1A, N9A–Cd2–N8 and O4B–Cd2–O3B angles formed by Cd1 and Cd2 centers-*N-bishydeten* ligands are 76.5(6)°, 90.9(6)°, 71.9(4)° and 91.9(8)°, respectively. Additionally, the Cd2–N7–C8, C8–Ag1–C7 and C7–N4–Cd1 bond angles are 170.0(4)°, 158.4(2)° and 165.6(4)°, respectively, while the Cd2–N11–C18, C18–Ag4–C20 and C20–N13–Cd1 bond angles are 171.4(4)°, 171.1(2)° and 167.2(5)°, respectively (Table S2). As a result, the significant deviation from linearity of the Cd–N–C and C–Ag–C angles leads to the formation of arc-shaped chains at the Ag1 and Ag4-centered respectively as seen from the Fig. 2 and Figure S5.

The anionic slices {[Ag(*μ*–CN–)_2_]_8_[Ag(CN)]_3_}^8−^ of the sandwich-type like structure are composed of the bridged Ag_8_(CN)_16_^8−^anions and the Ag_3_(CN)_3_ groups involved in argentophilic interactions. The argentophilic interactions observed between Ag(I) centers (*d*^10^–*d*^10^) are ligand-unbacked forces and have an essential role in complex stability and molecular clustering^57,58^. The Ag…Ag bond distances of the triple and quintet fragments of complex **4** have various values ranging from 3.12 to 3.23 Å which are below the sum of the vander Walls of two Ag atoms (3.44 Å)^59^. The available Ag…Ag interaction distances in complex **4** are 3.1216(7) Å for Ag1…Ag2, 3.2199(5) Å for Ag2….Ag3 and 3.2313(5) Å for Ag4….Ag5. On the other hand, the triple Ag1…Ag2…Ag3 [154.19(2)°] chain is significantly curved relative to the triple Ag2…Ag3…Ag2 [180.000°] and Ag3…Ag4…Ag3 [180.000°] chains (Fig. 2 and Table S2).

### EPR and magnetic properties

The powder EPR spectra of complex **1** containing Ag^+^ and Ni^2+^ ions at room temperature could not be observed. This situation may be because Ag^+^ ion is diamagnetic and Ni^2+^ ion does not signal because it has short relaxation times at room temperature. EPR spectrum analyzes of Ni^2+^ ion-containing complexes reveal that EPR signals cannot be obtained at room temperature, but very low-intensity peaks can be seen at very low temperatures^60^.

The powder EPR spectra of complex **2** are seen in Figure S9 in the Supplementary Material. The EPR spectra of **2** have been observed in the parallel and perpendicular components. The parallel peak is because the *dc* field is equal to the symmetry axis of the paramagnetic center. The values of *g*_⊥_ and *g*_//_ extracted from the powder spectrum of complex **2** are *g*_//_ = 2.210, *g*_⊥_ = 2.095, respectively. This spectrum belongs to Cu^2+^ ion (S = 1/2, I = 3/2). It can be inferred from the order of *g*_//_ > *g*_⊥_ > *g*_*e*_ (*g*_*e*_ = 2.0023, free electron *g* value) that, Cu^2+^ is located in distorted items (*D*_*4h*_) elongated along the ground state of the paramagnetic electron is $d_{{x^{2} - y^{2} }}$ (^2^B_1g_ state) and *z*-axis^61–64^. When the Lande *g* values of Cu^2+^ complexes containing tetracyanidometallate having neutral ligands are compared with those of complex **2**, it is noticed that *g* amounts are *g*_*//*_ > *g*_⊥_ > *g*_*e*_^33,34,48,49,65–68^_*.*_

The magnetic susceptibilities of **1** and **2** were recorded in the temperature of 10–300 K. The temperature dependence of magnetic (χ_m_) and χ_m_T are seen in Figures S10 and S11 (Supplementary Material) for both complexes. The variable temperature dependence of χ_m_ for both complexes were coordinated by the relation $\alpha + C/\left( {T - \theta } \right)$, which α is the temperature independent susceptibility (TIP)^69^. For **2**, the determined results are: $C = 0.588 \pm 0.0003 \;{\text{emuK/mol}}\;{\text{Oe}}$, $\alpha = 0.00027 \pm 0.000003 \;{\text{emu/mol}}\;{\text{Oe}}$ and $= - 4.9 \pm 0.009\;{\text{K}}$. As for **1**, the determined fitting results:$C = 2.56 \pm 0.0005 \;{\text{emu/mol}}\;{\text{Oe}}$, $\alpha = 0.00077 \pm 0.000004 \;{\text{emu/mol}}\;{\text{Oe}}$ and ${ } = { } - 0.6 \pm { }0.002{ }\;{\text{K}}$. The good magnetic moment for **1**, *µ*_*eff*_, was determined as 4.52 in Bohr magneton ($\mu_{B}$)^70^.

In this part, 10 K, for **1** and **2** could be very tiny antiferromagnetic interplay in the chemical structure, as observed in the insertion of Figures S10 and S11. The magnetic of **1** and **2** recorded a resemblance to tetracyanidometallate containing similar neutral ligands^33,34,48,49,65–68^. They even exhibited antiferromagnetic properties at low temperatures as in complexes **1** and **2**^33,48,49,65–68,71,72^.

### DNA topoisomerase I, DNA restriction endonucleases, and DNA binding studies

#### Determination of DNA topoisomerase I enzyme inhibitory activities

**Figure 4:**
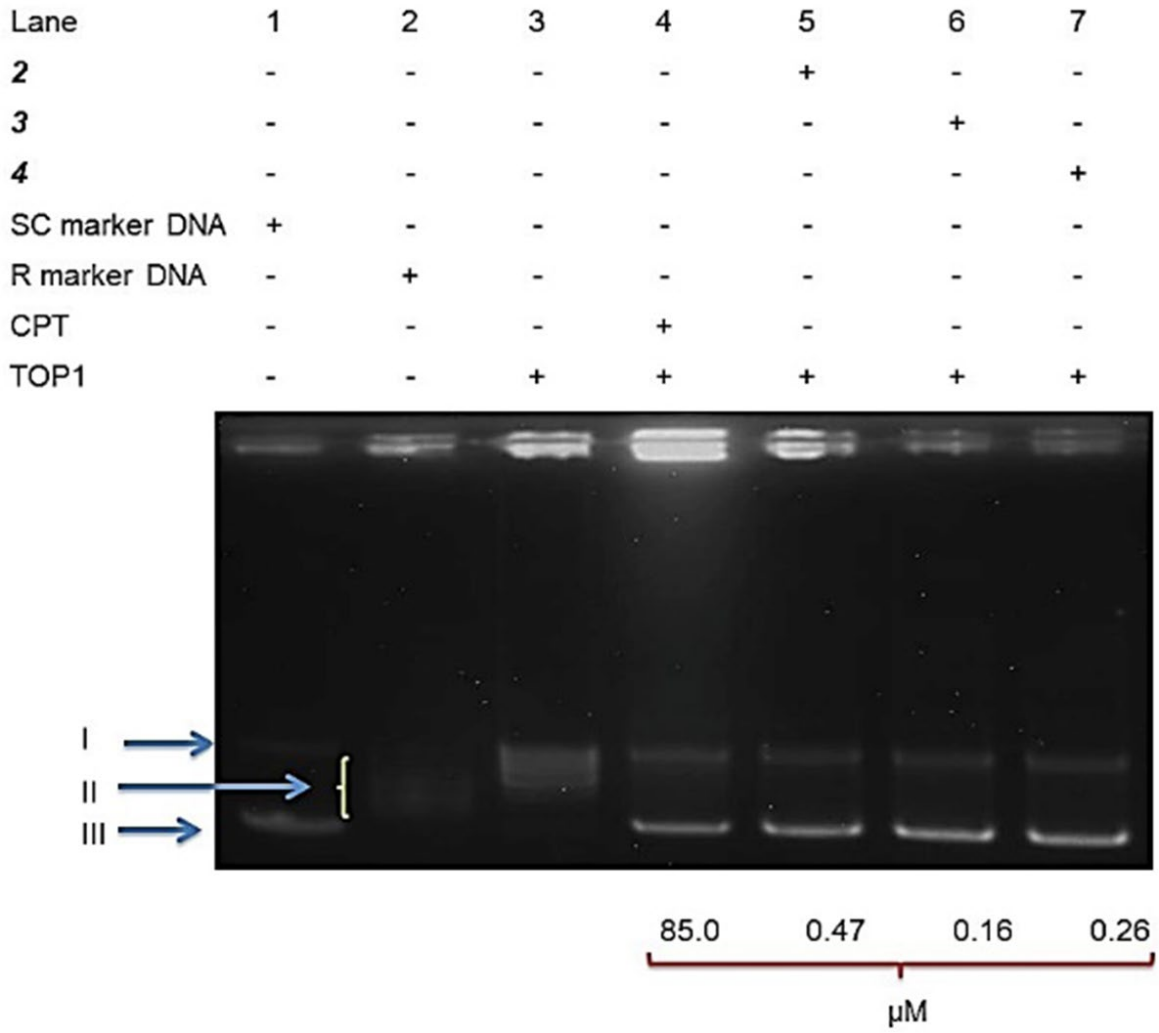
A DNA unwinding assay was performed with 2U TOP1, 250 ng pHOT-1 supercoiled DNA, and IC_50_ concentrations of **2**, **3** and **4**. The phases of the DNA molecule are denoted as II (Relaxed DNA), I (Nicked DNA), and, III (Supercoiled DNA). Lane 1 in this work is the supercoiled marker DNA; Also, Lane 2 represents the relaxed marker DNA molecule; Lane 3 in this study represents the negative standard (TOP1 + Supercoiled DNA); Finally, Lane 4 is the normal control (Camptothecin + TOP1 + Supercoiled DNA) and Lane 5–7 for this part represent test molecules over an IC_50_ value-concentration.

DNA topoisomerase that is an important target for anticancer agents are nuclear enzymes and alter the topological state of DNA molecule during the cell division and another cellular process such as replication and transcription^73,74^. Today, some topoisomerase inhibitor compounds like Camptosar, topotecan, and irinotecan have been utilized in clinical practice. Hence, to figure out the antiproliferative activities of these molecules includes inhibition of DNA topoisomerase I, we investigated the effects of these molecules on the recombinant act of topoisomerase I enzyme. The results showed that IC50 concentration of these compounds, for instance Camptothecin (Fig. 4), inhibited the DNA relaxation activity of topoisomerase I that they can be used as a new topoisomerase I inhibitor which acts through binding to topoisomerase I. The results of other studies also revealed that metal complexes bind to topoisomerase I and inhibited it^70,75,76^

#### Determination of DNA restriction endonucleases activity

In the presence of the **3** and **4**, DNA digestion was complete, and two bands were observed near the well at the top of the lanes (Lanes 1 and 2). Treatment of *Kpn*I and *Bam*HI with **3** and **4** failed to inhibit the restriction endonucleases activity of these enzymes. These results indicated that **3** and **4** did not bind to pTOLT plasmid DNA. However, the **2** caused the formation of two DNA bands (Lane 3) corresponding to the supercoiled and nicked DNA, that was observed in the undigested DNA (Lane 5) (Figure S12; Supplementary Material).

#### DNA binding study

**Figure 5:**
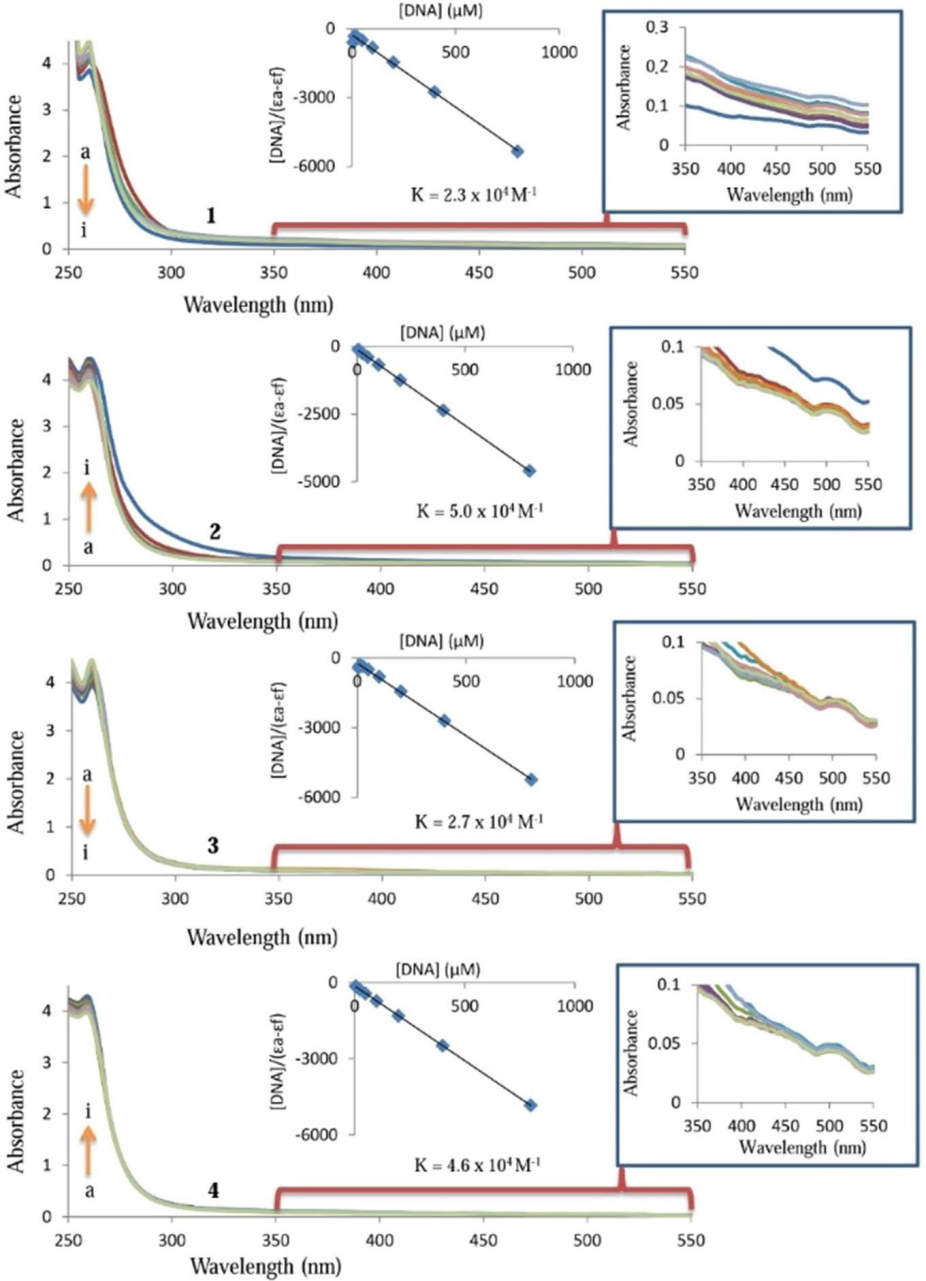
UV absorption spectra of 25 µM **1**, **2**, **3** and **4** in the absence (**a**) and presence of 6.25 µM (**b**), 12.5 µM (**c**), 25 µM (**d**), 50 µM (**e**), 100 µM (**f**), 200 µM (**g**), 800 µM (**i**) DNA.

UV–visible absorption spectroscopy technique has been utilized to work the interactions among DNA and **1**–**4**. The type of test molecule–DNA interactions and the binding constant (*K*_*b*_) may be evaluated by comparison of electronic spectra properties of the free test molecule and test molecule–DNA adduct. The binding constant of **1**–**4** with DNA can be obtained according to Wolfe–Shimmer equation, [DNA]/(ε_a_ − ε_f_) = [DNA]/(ε_b_ − ε_f_) + 1/[*K*_*b*_(ε_b_ − ε_f_)]. The *K*_*b*_ can be recorded from the ratio of the slope to intercept in the plot of [DNA] versus [DNA]/(ε_a_ − ε_f_), where ε_f_ and ε_a_ are the absorption coefficients of the **1**–**4** and its adduct, respectively^77^. Figure 5 represents the interaction of **1**–**4** with CT-DNA. The inset graph has the plot of [DNA] versus [DNA]/(ε_a_ − ε_f_) data which yielded the binding constant (*K*_*b*_) of 2.3 ± 0.17 × 10^4^ M^−1^ for **1**, 5.0 ± 0.24 × 10^4^ M^−1^ for **2**, 2.7 ± 0.19 × 10^4^ M^−1^ for **3**, and 4.6 ± 0.21 × 10^4^ M^−1^ for **4** (Fig. 5). There was a hyperchromic effect at the absorption bands of **2** and **4** indicating a strong interaction between them and DNA. However, the hypochromic effect was observed by the addition of increasing amounts of CT-DNA to **1** and **3**. The hypochromic effect of **1** and **3** contributed to the intercalation of **1** and **3** into the DNA base pairs and can be explained by decreasing the distance between DNA bases and intercalated **1** or **3**. Overall, the observed binding constant of the complexes against DNA and 5FU where binding constant were reported to be 5.73 × 10^4^ M^−1^ and 9.7 × 10^4^ M^−1^, respectively^59,60^.

In addition to UV–Visible absorption spectroscopy technique, the ethidium bromide exchange studies were also conducted to determine the binding affinity between **2**, **3**, **4** and DNA. The emission spectrum data EB bound to DNA in the presence and absence of **2**, **3**, and **4** are depicted in Figure S13 (Supplementary Material). The decreases in the fluorescence intensity of EB-DNA in the presence of **2**, **3**, and **4** implied that they might intercalate into a pair of the DNA. The quenching of EB to CT-DNA by the **2** is in harmony with the Stern–Volmer equation, which provides more evidence about the interaction between **2** and DNA, and is shown in Figure S13^77^. The *K*_*SV*_ value for the complex **2**, **3**, and **4** are 5.0 ± 0.32 × 10^3^ M^−1^, 13.7 ± 0.41 × 10^3^ M^−1^, 1.1 ± 0.12 × 10^3^ M^−1^, respectively. The reaction of the **3** with CT-DNA is more powerful than those of the **2** and **4** complexes.

### Stability study

The results of these molecules were conducted by utilizing a simple spectrophotometric assay. The molecules in physiological buffer (Phosphate buffered saline, 0.1 M, pH 7.4) were performed at regular intervals for 24 h. There were no changes in absorbance up to 24 h in complexes. Thus, the silver compounds proved to have the ability of high solution stability in a buffer (Table 2).

**Table 2:** UV–Vis spectrophotometric method.

Parameters	**1**	**2**	**3**	**4**
Linearity	0.98	0.96	0.95	0.97
Accuracy, % RSD < 2%	127.77	116.91	93.06	91.82
Precision, % RSD < 2%				
1.99	2.02	1.99	1.79	1.22
1.82	2.15	1.82	1.89	1.74
1.73	2.07	1.73	1.83	0.73
LOD	18.27	33.57	40.34	27.03
LOQ	55.38	101.75	122.24	81.93
% Error	12.00	11.47	19.83	9.17
Linearity range	1.95–250	1.95–250	1.95–250	1.95–250

This study was assessed using the absorbance values of eight diverse concentrations of the molecules within the same day and between various days. The repeatability, inner- and intra-day precision of the work displayed at since % RSD < 2% for the molecules. These molecules remained fairly stable (Figure S14 Table 2). The plots in measuring of the compounds were found to be linear in the scanning concentration range, and the linearity values of **1**, **2**, **3** and **4** were 0.95–0.98 for all (Table 2). The lowest amounts that could be detected (LOD) for **1**, **2**, **3** and **4** were 18.27, 33.57, 40.34 and 27.03 µM, respectively. The limits of quantification (LOQ) for **1**, **2**, **3** and **4** were found to be 55.38, 101.75, 122.24 and 81.93 µM, respectively, with a % RSD < 2%.

### Antiproliferative actions of the Ag(I) molecules

**Figure 6:**
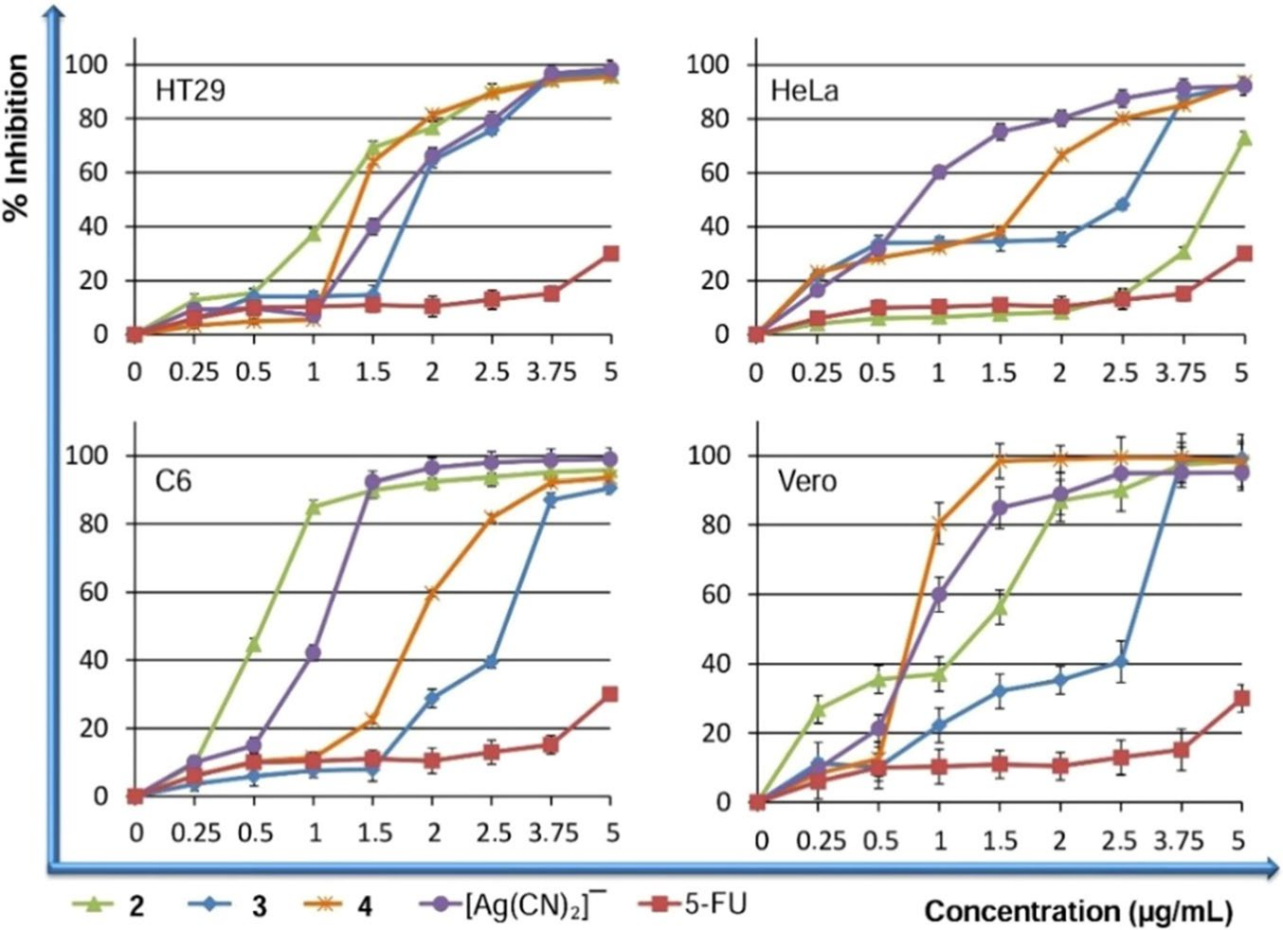
Effects of **2**, **3**, **4** and [Ag(CN)_2_]-on the proliferation of Vero cells, HeLa, C6, and HT-29. The growing cells were incubated with **2**, **3**, **4** and [Ag(CN)_2_] and the cell multiplication was obtained by the BrdU Elisa method.

The antiproliferative activities of Ag(I) molecules and the starting molecules of the parent molecules, *N-bishydeten* and [Ag(CN)_2_]^−^ compound, on cells, were monitored utilizing by the BrdU Cell Proliferation Assay (BCPA) (Fig. 6). To determine whether selectively killed the cancer agents in the absence of being detrimental to the standard cells, we determined the antiproliferative actions of our molecules towards colon, cervical, a normal cell line (Vero), and brain cancer cell lines. BCPA test effects implied that 2 (0.87–3.64 µM), 3 (2.37–3.34 µM), 4 (0.48–0.63 µM) and [Ag(CN)_2_]^−^ (5.08–5.63 µM) ligand disclosed very high antiproliferative effects on these cells, while ligand, *N-bishydeten*, seen no antiproliferative effects towards cancer with the same administrative dose (data not shown). Antiproliferative activities of complex **2** were higher on HT29 (0.87 ± 0.09 µM) and C6 (0.95 ± 0.09 µM) cells in comparison to Vero cells (Fig. 6 and, Table 3). This text means that complex **2** has an interesting selectivity towards cancer cells. Tumor specificity index (TSI) and IC_50_ amounts to be used in consequent studies were recorded by performing the BrdU ELISA method, and these are given in Table 3.

**Table 3:** IC50 values and tumor specificity index.

Compounds	IC50 (µM)				Tumor specificity index		
	HeLa*	HT29*	C6*	Vero*	HeLa	HT29	C6
**2**	3.64 ± 0.42	0.87 ± 0.09	0.95 ± 0.09	2.81 ± 0.32	0.77	3.23	2.96
**3**	3.19 ± 0.37	2.37 ± 0.32	3.34 ± 0.34	3.06 ± 0.35	0.96	1.29	0.92
**4**	0.61 ± 0.09	0.48 ± 0.08	0.60 ± 0.09	0.63 ± 0.09	1.03	1.31	1.05
5FU	275.68 ± 17	258.46 ± 21	217.48 ± 19	258.46 ± 21	0.94	1.00	1.19
Cisplatin	230.41 ± 15	152.57 ± 14	207.51 ± 23	234.83 ± 19	1.02	1.54	1.13
[Ag(CN)_2_]¯	5.13 ± 0.87	5.18 ± 0.93	5.08 ± 0.89	5.63 ± 0.96	1.10	1.09	1.11

*Values are given as the mean ± SD of three experiments and r^2^ = 0.91 to 0.98. Significant at *P* < 0.05.

The find tumor specificity index results divided by the sum of the IC_50_ amounts from normal cells (Vero) to the sum of the IC_50_ values of the cancer cells (C6, HeLa, HT29) (Table 3). Molecule **2** recorded the best selectivity for the HT29 (3.23 TSI) and C6 (2.96 TSI) cells over the Vero cells while compounds **3** (1.29 TSI) and **4** (1.31 TSI) displayed poor selectivity for the HT29 cells. The cell proliferation results disclosed that the Ag(I) molecules were remarkably more antiproliferative than cisplatin and 5FU (Fig. 6)^30–34,58^.

### Cytotoxic profile of the Ag(I) compounds

LDH test results revealed that **2**, **3**, and **4** exhibited the identical cytotoxic effects as the 5FU, Indeed, bridging ligand [Ag(CN)_2_]^−^, caused greater cytotoxicity than positive control on some cell lines (Figure S15; Supplementary Material). It is observed that [Ag(CN)_2_]^−^ is a highly toxic molecule towards both standard and tumorigenic cells. However, it was found to have a limited effect while examining the contribution of [Ag(CN)_2_]^−^ to the antiproliferative and cytotoxic activities of our compounds. As seen in Fig. 6 and Figure S15, the antiproliferative and cytotoxic activity of **2**, **3**, and **4** were lower than bridging ligand, recording that the cytotoxicity of [Ag(CN)_2_]^−^ reduced to safe levels in Ag(I) compounds. All compounds and 5FU or cisplatin (9–11%) tested and also were found to be moderately cytotoxic against HT29 cells. However, an important reduce in cytotoxicity was obtained for **2** when their activity on cells was evaluated. The IC50 values obtained from our compounds (0.48–3.64 μM) were lower than those of 5FU (275.68–258.46 μM) or those of cisplatin (152.57–234.83 μM) (Table 3). However, it is necessary to conduct in vivo studies in order to determine the real cytotoxic effect of these compounds. An ideal anticancer drug would exterminate cancer cells without disturbing normal cells and has cytostatic profiles that can activate apoptosis^78^.

### Determination of the apoptotic effect of the Ag(I) complexes by DNA laddering method

**Figure 7:**
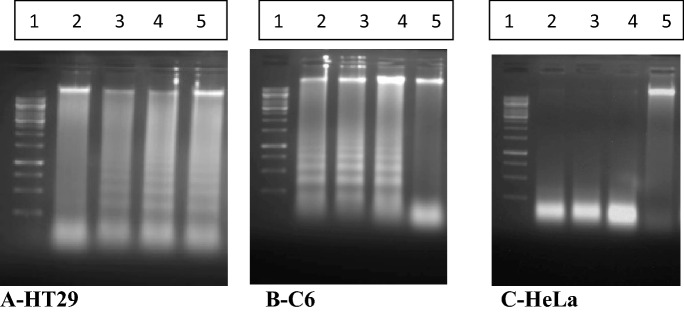
A representative outcome shows the bands of **2**, **3** and **4** on internucleosomal DNA fragmentation act in cancer cells. By growing C6, HeLa, and HT29 cells were incubated with the molecules at 37 °C for overnight, DNA molecule was isolated in this part, and DNA fragmentation was visualized by agarose gel electrophoresis. All compounds induced DNA fragmentation. **A-HT29**: 1, DNA standard; 2, HT29 Control; 3, HT29 + **2**; 4, HT29 + **3**; 5, HT29 + **4**. **B-C6**: 1, DNA standard; 2, C6 + **2**; 3, C6 + **3**; 4, C6 + **4**; 5, C6 Control. **C-HeLa**: 1, DNA standard; 2, HeLa + **2**; 3, HeLa + **3**; 4, HeLa + **4**; 5, HeLa Control.

DNA laddering revealed the **2**, **3**, and **4** induced the organization of DNA fragmentation in cancer cells in comparison to the standard cells (Fig. 7). Here, appearances of apoptotic morphology and DNA fragmentation may be a result of the activation of the extrinsic apoptotic pathways, including Ca^2+^ dependent endonucleases. Apoptosis assay is determined by controlling cell death which included cleavage of DNA molecule into regular fragments. In this part, we observed that our molecules could act through containing apoptosis on some cells. More studies were conducted to obtain the antiproliferative and apoptotic potentials of Ag molecules which are consistent with this work^70,75,75,79,80^.

### The apoptotic effect of the Ag(I) molecules at the single cell level

**Figure 8:**
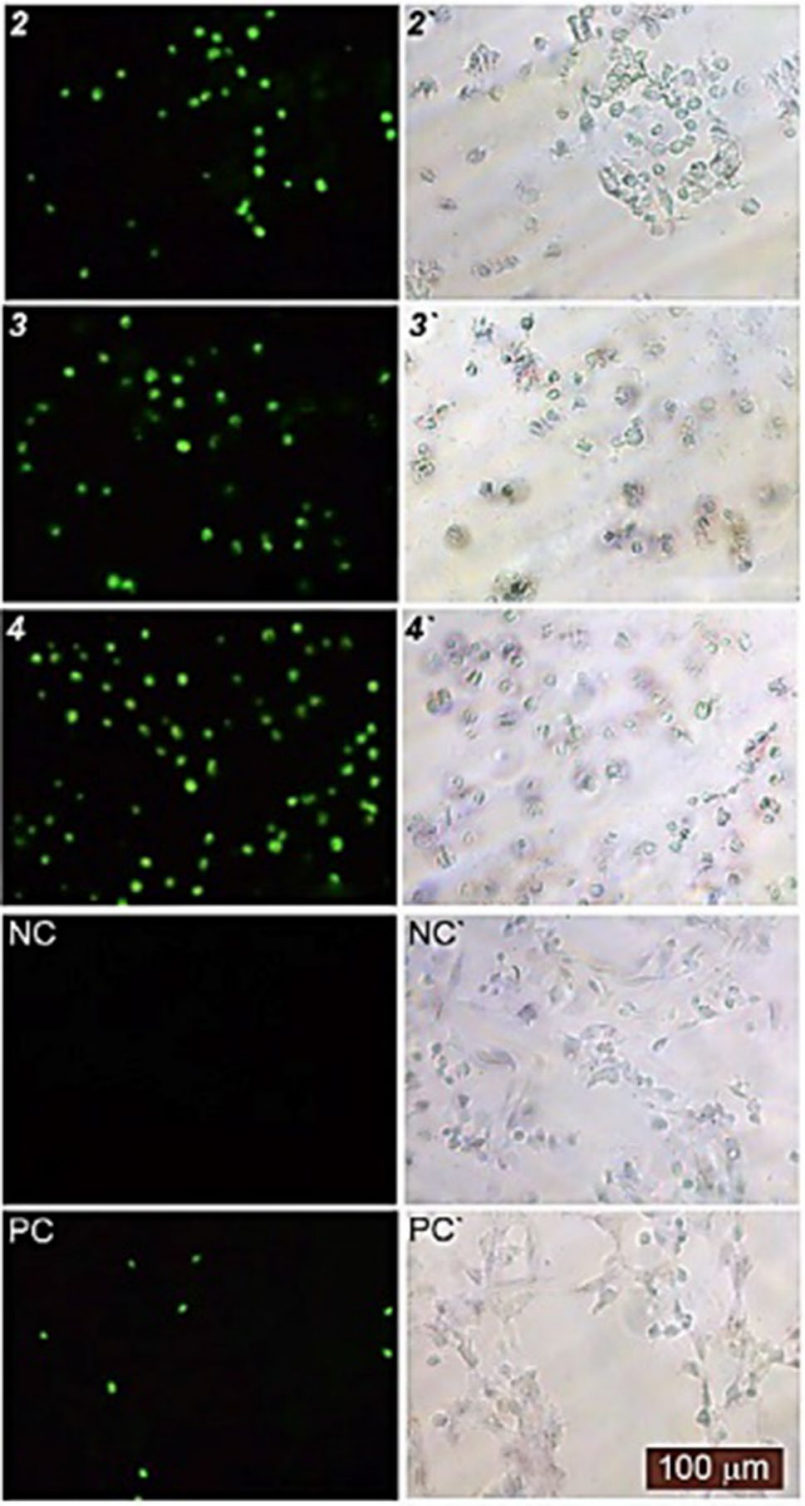
Phase-contrast and Fluorescence and image of the HT29 cancer cell line examined by TUNEL assay. TUNEL-positive cell nuclei in brilliant green were observed under a fluorescence (**2**, **3**, **4**, NC and PC) and phase-contrast microscope (2ʹ, 3ʹ, 4ʹ, NCʹ, and PCʹ). **2**, **3** and **4** treatments (*NC* negative control, *PC* positive control).

Ag(I) compounds were found to exhibit a possess pharmacological effect and vigorous antiproliferative property. We have evaluated the apoptotic effect of the molecules on HT29 cells utilizing the TUNEL method as an immunohistological study to reveal their mechanism of action on cells. The apoptotic activity of the Ag(I) compounds was examined in human colon cancer cells, HT29. The TUNEL method stressed the formation of the apoptosis in the HT29 cells with concentrations of 0.87 ± 0.09 μM for **2** and 2.37 ± 0.32 μM for **3** and 0.48 ± 0.08 μM for **4** for 24 h. In Fig. 7, HT29 cells treated with Ag(I) compounds caused green fluorescence, indicating fragmented DNA in apoptotic cells. As illustrated in Fig. 8, TUNEL results depicted that **2**, **3**, and **4** significantly triggered apoptosis on HT29 cells. The Ag(I) compounds clearly exhibited better apoptotic features rather than positive control treated with DNase I. Generally, in Fig. 7, Ag(I) compounds led to a significant enhancement of TUNEL-positive cells, in contrast to the control group. These findings indicated that **2**, **3** and **4** inhibit cell proliferation by inducing apoptosis desiring property for anticancer agents. As shown by similar studies, Ag(I) complexes exhibiting TUNEL positive activity acted as an apoptotic factor against cancer cells^81^. In previous studies, we demonstrated that nine good-soluble Ag(I) compounds containing different ligand and Ni, Cu, Zn, and Cd metal salts complex acting as an apoptotic agent against HT29 cells, exhibit TUNEL positive activity^15,19,35^. In a similar study performed with TUNEL assay, 2-Mercaptobenzothiazole (MBT) complexes of Ag(I) exhibited apoptotic features against A549 cells^82^. Overall, these results highlighted that these Ag(I) compounds with strong apoptosis-inducers may have a strong antiproliferative effect on colon cancer in vitro.

### BSA binding study

**Figure 9:**
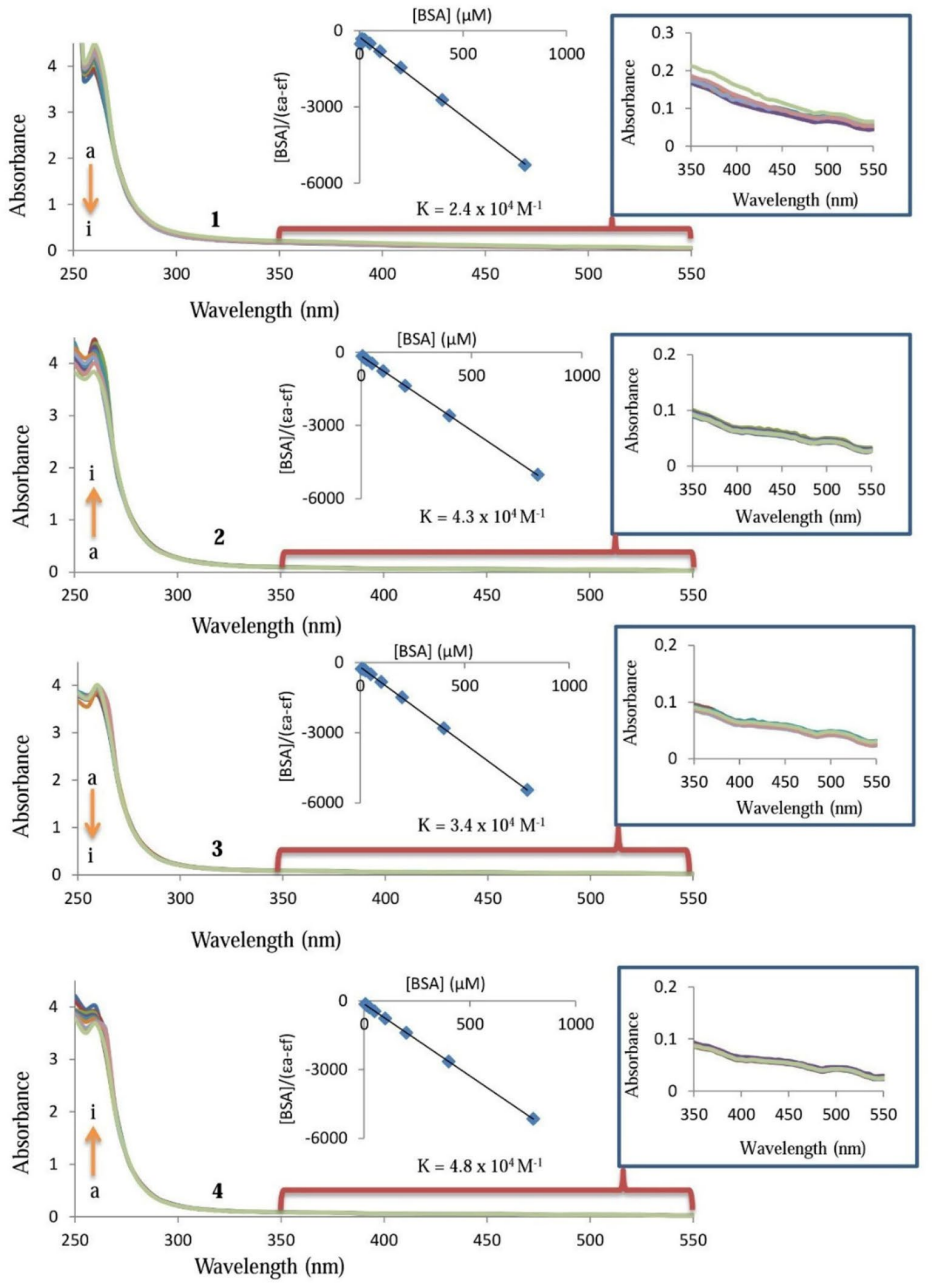
UV absorption spectra of 25 µM **1**, **2**, **3** and **4** in the absence (**a**) and presence of 6.25 µM (**b**), 12.5 µM (**c**), 25 µM (**d**), 50 µM (**e**), 100 µM (**f**), 200 µM (**g**), 800 µM (**i**) BSA.

UV–Visible absorption spectroscopy technique was also used to study interactions between BSA and **1**–**4**. The absorption spectra of the BSA (6.25–800 μM) in the presence and absence of the **1**–**4** (25 μM) are displayed in Fig. 9. The some results of [BSA]/(ε_a_ − ε_f_) = [BSA]/(ε_b_ − ε_f_) + 1/[*K*_*b*_(ε_b_ − ε_f_)] in Fig. 9, used and also obtained. The binding constants for the **1**–**4** were found as 2.4 ± 0.22 × 10^4^ M^−1^, 4.3 ± 0.29 × 10^4^ M^−1^, 3.4 ± 0.23 × 10^4^ M^−1^ and 4.8 ± 0.29 × 10^4^ M^−1^, respectively. In Fig. 9, the increase in BSA concentrations leads to a change in the absorption of the complexes resulting in hypochromism for **1** and **3** and hyperchromism for **2** and **4**. These results suggested that an interaction exists between these complexes and BSA similar to the DNA binding mode of the complexes. In addition, the molecules caused an upward trend in the BSA absorbance and exhibited a slight redshift, indicating the presence of van der Waals or hydrogen bonds between BSA and them.

### The effect of the Ag(I) molecules on HeLa cell migration

The capacity of the cancer cell migration is an excellent target for anticancer agents because tumor cells may escape from the apoptosis mechanism by using its migration capability. Successful cancer treatment involves both inhibition of cancer proliferation and suppression of the migration effect as plenty of cancer cells exhibit potent cell growth and invasive behavior. Ag(I) compounds at 50% maximal inhibitory concentration (IC_50_) decelerated HeLa cell migration and enhanced apoptotic stress (Figure S16). The **2**, **3** and **4** can also restrict the level of development of HeLa cells, indicating they could be entered to preclinical trials.

Ag(I) compounds at 30% maximal inhibitory concentration (IC_30_) managed to inhibit tumor cell migration with low cytotoxicity (data not shown). In addition, this cytotoxic ability of Ag(I) compounds at 20% maximal inhibitory concentration (IC_20_) allowed the suppression of cell migration without damaging the cell membrane at non-toxic concentrations (data not shown). After 72 h of incubation, the migration rate of the treated cells failed to fill the gap (Figure S16; Supplementary Material, Day 2), and untreated HeLa cells accomplished to form throughout the gap. While untreated HeLa cells filled 100% of the gap, compounds **2**, **3**, and **4** managed to spread to 9%, %11, 15% of the gap, respectively (Figure S16; Supplementary Material, Day 2). In addition, the media with Ag(I) compounds were replaced with fresh media following the 72-h incubation, but the HeLa cells could not fill the gap (data not shown). That is, the Ag(I) compounds may exhibit cytostatic effect by inhibiting cell growth and multiplication. The result was consistent with that of the other studies on Ag(I) complexes containing different ligands^73,74,78,80,81,83–87^.

### The effect of the Ag(I) complexes in the morphology of the cells

As shown in Figure S17 (Supplementary Material), obvious morphological changes were recorded in the treated cells when in comparison to the untreated cells. Each cell line exposed to the compounds exhibited cytoplasmic blebs, shrinkage, anomalous globular structure; these are all hallmarks of the apoptotic cell death. The results in Figure S17 have also displayed the normal structure of the most control cells. Most of the treated cells had an abnormal fibroblast-like appearance and were detached from the plate surface. Moreover, the cells began to separate from one another and to appear smaller. These situations were consistent with the outcomes of TUNEL methods, and this finding was similar to those of previous studies^83–87^. According to information found in literature^88–90^, the appearance of the cells treated with **2**, **3** and **4** clearly indicated the the quality and the number of cells in the flask monolayer were reduced.

### IHC investigation of slides treated by Ag(I) molecules

Immunohistochemistry staining was found to reduce the expression of Bcl-2 and increase the expression of P53 in Ag(I) complexes-treated the cells, which emphasizes the apoptotic effects of these molecules (Figures S18 and S19). These findings are agreeable with those of similar works^91^. The results also revealed that Ag(I) complexes treated cells significantly reduced the expression of cytokeratins (CK20 and CK7) releasing from proliferating or apoptotic cells. This condition can be associated with the reduced metastatic capability via an anti-migratory potential of these molecules due to the influenced intermediate filament (IF) proteins.

### Antibacterial activity

The increasing amount of experimental data available in the literature show that there are some powerful links among the pathogen bacterial flora (i.e., septicemia) or the opportunistic agents (i.e., pneumonia infections) and certain cancer kinds like urogenital, cervical, stomach cancers, liver, and lymphoproliferative disturbances^70^. Therefore, the pathogen bacterial flora and the opportunistic agents may be taken into consideration both in the management of cancer patients and in individual susceptibility to cancer^80^. Indeed, dual acting factors with antimicrobial and antiproliferative potentials can result in improved therapeutic efficacy for cancer cell patients or reduced cancer predisposition. In light of this information, the antimicrobial activities of **1**–**4** were also tested *against* four gram-positive bacteria and five gram-negative bacteria. The experiments were conducted in triplicate to prevent possible errors, and SCF [Sulbactam (30 µg) + Cefoperazone (75 µg)] was used as a standard drug^53,92,93^.

Results were **4** > **2** > **3** > SCF > **1** > KCN for antibacterial activities while for *S. enteridis,* and *S. gallinarum* were **4** > SCF > **1** > KCN sequence of antibacterial effects (Tables 4 and 5). The bacterial inhibition sites of complexes **1**–**4** are shown in Figures S20, S21 and S22 ( Figures S20, S21 and S22 Table S3; Supplementary Material). The values of 36 and 37 mm made it obvious that the antibacterial effect of type of **4** was the strongest among all.

**Table 4:** Antibacterial activity of **1**–**4** (105 µg/disc).

Microorganisms	Compounds and inhibition zones (mm)					
	SCF	KCN	**1**	**2**	**3**	**4**
**Gram-positive bacteria**						
*S. aureus* ATCC29213	29	–	18 ± 0.58	37 ± 1.0	25 ± 0.58	30 ± 1.0
*B. subtilis* ATCC6633	19	–	14 ± 1.53	27 ± 0.58	31 ± 1.0	34 ± 0.58
*B. cereus* DSM 4312	30	–	20 ± 1.0	30 ± 0.58	34 ± 0.0	36 ± 1.0
*St. pyogenez* ATCC176	20	–	15 ± 0.0	32 ± 0.58	29 ± 0.58	26 ± 0.0
**Gram-negative bacteria**						
*E. coli* 111	21	–	19 ± 0.58	32 ± 0.0	30 ± 0.58	29 ± 0.58
*E. aerogenes 2924*	31	–	15 ± 1.0	22 ± 1.0	20 ± 1.0	25 ± 1.0
*S. gallinarum*	30	–	17 ± 0.58	35 ± 1.0	35 ± 0.58	37 ± 1.0
*P. aeruginosa* ATCC9027	15	–	–	30 ± 1.0	30 ± 1.0	30 ± 0.58
*S. enteridis* ATCC13076	22	–	18 ± 0.58	30 ± 0.58	33 ± 0.58	36 ± 1.0

SCF, sulbactam (30 µg) + cefoperazone (75 µg), as a positive control.

KCN, potassium cyanide, as a negative control.

**Table 5:** Minimum-inhibitory concentrations (MIC, in mg/mL) of **1** and **4**.

Microorganisms	KCN	**1**	**4**	SCF
**Gram-positive bacteria**				
*S. aureus* ATCC29213	–	62.50 ± 36.08	62.50 ± 0.0	250
*B. subtilis* ATCC6633	–	62.50 ± 0.0	31.25 ± 0.0	500
*B. cereus* DSM 4312	–	62.50 ± 18.04	62.50 ± 18.04	500
*St. pyogenez* ATCC176	–	15.62 ± 9.02	31.25 ± 9.02	500
**Gram-negative bacteria**				
*E. coli* 111	–	62.50 ± 0.0	31.25 ± 0.0	250
*P. aeruginosa* ATCC9027	–	62.50 ± 18.04	62.50 ± 0.0	1000
*E. aerogenes* ATCC2924	–	15.62 ± 0.0	125 ± 53.40	62.50
*S. gallinarum*	–	31.25 ± 0.0	125 ± 0.0	62.50
*S. enteridis* ATCC13076	–	62.50 ± 17.97	62.50 ± 0.14	1000

SCF, sulbactam (30 µg) + cefoperazone (75 µg), as a positive control.

KCN, potassium cyanide, as a negative control.

In this study, molecules **4** and **1** were subjected to MIC, and the findings profiles are submitted in Table 5. Sulbactam (30 µg) + Cefoperazone (75 µg) (105 µg/disc), were used as the standard and investigated by the Serial microdilution method to obtain MIC values in Mueller–Hinton Broth for the antibacterial test. The inhibition zones and MIC amounts for strains for **4** and **1** were recorded in the range of 15–37 mm and 15.62–125 μg/mL, respectively (Tables 5 and 4). Four types of gram-positive bacterial strains (*St. pyogenez, B. subtilis, B. cereus, S. aureus*) and five types of the gram-negative (*S. enteridis, E. aerogenes, P. aeruginosa, E. coli, and S. gallinarum*) were sensitive to **4** and **1**. For the **4**, the MIC and inhibition zones values of the bacterial strains were found as 31.25–125 μg/mL and 25–37 mm, respectively (Tables 5 and 4). In Table 5, molecule **1** (MIC: 15.62, 15.62, 31.25 µg/mL, respectively) exhibited better activities than molecule **4** and the standard for *St. pyogenez*, *E. aerogenes, and S. gallinarum* bacteria.

## Conclusion

In this present study, four different complexes were synthesized using Ni^2+^ **(1)**, Cu^2+^ **(2)**, Zn^2+^ **(3)**, Cd^2+^ **(4)**, K [Ag(CN)_2_], and *N- bishydeten* and characterized by some advanced analytical techniques. Complex **4** consisting of [Cd(*N-bishydeten*)]4[Ag(CN)_2_]8[Ag(CN)] has a sandwich-type layered structure verified by the crystal method. In addition, the complexes were studied for their pharmacological properties, and they exhibited very strong anticancer **(2–4)** and antimicrobial activities **(1–4)**. The compounds, especially **2**, possessed more selective cytotoxic activity than the positive control against cancer cells, particularly HT29. The interaction of **1–4** with CT-DNA and BSA was shown with respect to the spectral changes in their absorbance, and their binding affinity was found to be very similar to the currently used anticancer agents such as cisplatin and 5FU. In future studies, we will try to improve the amount and functionality of our Ag(I) complexes using different ligands, metal salts, and new methods. Since the in vitro biological properties of these Ag(I) complexes can be used mainly against some cancer cell lines, in vivo anticancer study is very important to reveal the mechanism of action. In summary, our results show that these molecules are potentially valuable drug candidates and are suitable for further pharmacological testing.

## Supplementary information

Supplementary file1 (DOCX 5681 kb)

## Data Availability

X-ray graphic files in CIF format for 4 and crystallographic results for the chemical structure reported here have been deposited with the Cambridge Crystallographic Data Centre as supplementary data, CCDC Nos. 1519618. Copies of the data can be obtained through application to CCDC, 12 Union Road, Cambridge CB2 1EZ, UK. (Fax: + 44 1223 336033 or e-mail: deposit@ccdc.cam.ac.uk or at https://www.ccdc.cam.ac.uk).
