# Supplementary material for: Metalo components exhibiting significant anticancer and antibacterial properties: a novel sandwich-type like polymeric structure
Source: Sci Rep. 2020 Jul 27;10:12472. doi: 10.1038/s41598-020-69416-x (PMC7385626; doi:10.1038/s41598-020-69416-x)
Supplement: Supplementary file 1 — Supplementary file1 (DOCX 5681 kb) [file 41598_2020_69416_MOESM1_ESM.docx]

**Supplementary Material**

**Metalo components exhibiting significant anticancer and antibacterial properties: A novel sandwich-type like polymeric structure**

Ahmet Karadağ^a,^*, Nesrin Korkmaz^b^, Ali Aydın^c^, Hüseyin Akbaş^d^, Şaban Tekin^e,f^, Yusuf Yerli^g^ and Fatih Şen*^h^

^a^Department of Chemistry, Faculty of Arts and Sciences, Yozgat Bozok University, 66200 Yozgat, Turkey

^b^Faculty of Science, Department of Biotechnology, Bartın University, 74100 Bartın, Turkey

^c^Department of Basic Medical Science, Faculty of Medicine, Bozok University, 66200 Yozgat, Turkey

^d^Faculty of Art and Science, Department of Chemistry, Tokat Gaziosmanpaşa University, 60250 Tokat, Turkey

^e^TÜBİTAK MRC Genetic Engineering & Biotechnology Institute 41470 Gebze, Turkey

^f^Faculty of Medicine, Department of Basic Medical Sciences, Medical Biology, University of Health Sciences, 34668 Istanbul, Turkey

^g^Art and Science Faculty, Physics Department, Yıldız Technical University, 34220 Istanbul, Turkey

^h^Sen Research Group, Biochemistry Department, Faculty of Arts and Science, Dumlupınar University, Evliya Çelebi Campus, 43100 Kütahya, Turkey.

E-mail address: ahmet.karadag@bozok.edu.tr; fatih.sen@dpu.edu.tr

**General materials and methods**

AgNO_3_, KCN, CuCl_2_.2H_2_O, NiCl_2_.6H_2_O, ZnCl_2_, CdSO_4_.8/3H_2_O and *N,N*-bis(2-hydroxyethyl)-ethylenediamine (C_6_H_16_N_2_O_2_) were obtained and utilized without subsequent purification. Also, elemental methods (C, H and N) were carried out utilizing a LECO CHNS-932 analyzer. IR method was measured in the 4000-400 cm^-1^ region with a Jasco 430 FT-IR Spectrometer in KBr pellets. High-resolution mass spectrometry measurements were determined with using an Agilent 6530 Accurate-Mass with Q-TOF LC/MS system. The thermal procedures were investigated on Perkin Elmer Diamond TG/DTA Thermal Analysis Instrument in nitrogen atmosphere with a heating rate of 3 or 10 ^o^C min^-1^ and 5-10 mg sample. The 10-300 K measurement was carried out on a Quantum Design PPMS assay. χ-T plot was determined under the constant magnetic field of 5 kOe. Also, magnetic value was corrected for the sample diamagnetic contribution.Indeed, the EPR powder assays were recorded with a Bruker EMX X-band spectrometer about 20 mW microwave power and 100 kHz magnetic field modulation.

**Crystallography**

For determination of crystal structure of this stufy, the single-crystal of [Cd(*N-bishydeten*)]_4_ [Ag(CN)_2_]_8_[Ag(CN)]_2_ (**4**) was utilized for data collection on procedure of Rigaku R-AXIS RAPID-S imaging plate diffractometer. The graphite-monochromatized Mo K_α_ radiation (*λ*=0.71073 Å) and oscillation scanning technique with Δ*ω*=5˚ were used for data collection. The lattice parameter was determined by the least-squares procedures on the reflections with *F*^2^>2*σ*(*F*^2^). Also, correction act for polarization and Lorentz effects and cell refinement were determined using CrystalClear (Rigaku/MSC Inc., 2005) software ^44^. The structure was solved by direct procedures with using SHELXS-97 assay and refined by a full-matrix least-squares method utilizing the program SHELXL-97. Hydrogen’s attached to nitrogen and carbon atoms were positioned and also for this part, refined utilizing a riding model. Structure refinement parameters and Crystal data and for **4** are presented in Table 1.

**Table S1** Crystal data and structure refinement of **4**.

| Empirical formula | C_42_H_64_N_26_O_8_Ag_10_Cd_4_ |
| --- | --- |
| Formula weight / F(000) | 2589.49/ 2448 |
| Temperature | 293(2) |
| Crystal size (mm) | 0.52 x 0.31 x 0.11 |
| Crystal system / space group | Monoclinic / P2_1_/c |
| *a* (Å) | 22.4405(4) Å |
| *b* (Å) | 7.24325(14) Å |
| *c* (Å) | 28.4605(5) Å |
| (^o^) | 90 |
| *β* (^o^) | 128.7976(11) |
| *γ* (^o^) | 90 |
| V (Å^3^) /Z | 3605.36(11) / 2 |
| Density_calcd._(gcm^-3^) | 2.385 |
| Abs. coeff. *μ* (mm^-1^) | 3.862 |
| *θ* range (^o^) | 1.16– 26.81 |
| Index ranges | -28 ≤ *h* ≤ 28, -9 ≤ *k* ≤ 9, -35 ≤ *l* ≤ 36 |
| Reflections collected | 77429 |
| Independent reflections | 7673 [*R_int_*=0.0522] |
| R incides (all data) | *R_1_*=0.0341, *wR_2_*=0.0823 |
| Absorption correction, *T_min_*/ *T_max_* | Integration, 0.087/0.517 |
| w=1/[σ^2^(Fo^2^)+( 0,0372P)^2^+4,5081P] | P=(Fo^2^+2Fc^2^)/3 |
| S, (Δ/σ)_max_ | 1.039 / 0,001 |
| Δ*ρ*_max_, Δ*ρ*_min_ (eÅ^-3^) | 0.939 / -0.820 |

**Anticancer Studies**

The BSA/DNA binding feature of the molecules was evaluated using fluorimetric, UV–Visible spectroscopy, and enzyme inhibition restrictions. Inhibition of cytotoxicity and cell proliferation by **2−4** in HT29, C6, HeLa, and Vero cell was assessed with a LDH cytotoxicity assay and cell proliferation ELISA BrdU assay, respectively. The process of **2−4** in apoptosis was determined by terminal deoxynucleotidyl transferees dUTP nick end labeling method, immunohistochemistry, DNA topoisomerase I inhibition assay, and DNA laddering assay.

**Cell Culture**

The antiproliferative activity of the **2**, **3**, **4**, [Ag(CN)_2_]^−^ and 5FU against various cell lines (HeLa ATCC CCL-2, C6 cells ATCC CCL-107, HT29 ATCC HTB-38, and Vero ATCC CCL-81) were preserved and maintained in Dulbecco’s modified eagle’s medium, supplemented with %2 antibiotic (Streptomycin-Penicillin) solution and 10% fetal bovine serum.

**BrdU Cell Proliferation Assay (BCPA)**

The cell suspension including 5×10^3^ cells in 100 µL was supplied into the well cell plates (96-well cell). The therapies of these cells with **2**, **3**, **4** and [Ag(CN)_2_]^−^ in DMSO (max 0.1% DMSO) and 5-Florouracil (5 FU) molecule in DMSO (max 0.1% DMSO) separately at concentration 0.25, 0.50, 1.00, 1.50, 2.00, 2.50, 3.75, and 5.00 µg/mL were carried out. The last volume was adapted to 200 µL by mixture and DMEM and also incubated for **24** h at 37°C with 5% CO_2_. The antiproliferative activities of the **2**, **3**, **4** and [Ag(CN)_2_]^−^ were determined with using BrdU Cell proliferation ELISA Kit conforming to manufacturer’s assay.

**Calculation of IC_50_ and % inhibition**

IC_50_ values the concentration of a factor which required for other method of in vitro. In cell proliferation method results were observed as inhibitory effect of control and test substances. The % inhibition was obtained conforming to the formula: % inhibitions [1-(Absorbance of Treatments/ Absorbance of control) × 100].Indeed, the half maximal inhibitory effect concentration (IC_50_) of the control and test molecules was find using method of XLfit5 software (IDBS) and expressed in µg/mL at 95 % confidence intervals.

**Lactate Dehydrogenase (LDH) Cytotoxicity Assay**

The cytotoxic effect of the **2**, **3**, **4**, [Ag(CN)_2_]^−^ and 5FU on the HT29, HeLa, C6, and Vero cells was conducted the calorimetric LDH Cytotoxicity effect Kit based on the evaluation of LDH activities created from the damaged cell membranes into the supernatant conforming to manufacturer’s instructs. 5x10^3^ cells in 100 µL was conveyed into the well cell plates (96-well cell) and also treated with IC50 concentrations of **2**, **3**, **4** and [Ag(CN)_2_]^−^ as described above for **24** h at 37°C with 5% CO_2_. LDH activities were measurement at 492-630 nm using a microplate reader. The percentage cytotoxicity was find with using the equation, high control / low control - experimental value - low control x 100, where experimental value is the test-molecule treated cells, and high control is Triton X-100 treated cells, and low standard or control (spontaneous LDH enzyme) is the untreated cells.

**Apoptotic profiles by DNA laddering method**

DNA fragmentation effect of the **2**, **3** and **4** was displayed conforming to the procedure of Gong with several modifications. Briefly, 7.5x10^5^ cells were determined into 25 cm^2^ culture flasks, and obtained with IC_50_ values of **2**, **3** and **4** for **24** h at 37 °C with 5 % CO_2_. Treated cell was harvested using a plastic scraper, and these fixed with 70 % ethanol, and also incubated for 48 hours. The ethanol amount was removed by centrifugation and air drying. The cell pellet was resuspended in 55 μL phosphate-citrate buffer (consisting of 192 parts of 0.2 M Na_2_HPO_4_ and 8 parts of 0.1 M citric acid, pH 7.8), and incubated at 37°C for 30 min in a shaker incubator. A 45 μL of supernatant was transferred to a microsantrifuge tube, mixed with 5 μL 0.25% Tween20 solution and 5 μL RNase A enzyme solution and also incubated for 30 min in a shaker incubator. The following stage 5 μL proteinase K was added to each tube and incubated at 37 °C for 10 min. Finally, 4 μL of 6x loading buffer was added to entire contents in a microsantrifuge tube and loaded to 1.5% agarose gel containing 1.0 μg/mL ethidiumbromide and electrophoresed at 200 mA for 45 min. DNA laddering in the gels was photographed using a gel documentation system.

**Apoptotic potential by TUNEL assay**

In situ finding of apoptosis was conducted utilizing a TUNEL method kit conforming to the manufacturer’s mechanism. HT29 cells (30.000 cells/chamber slide section) were conveyed in poly-L-lysine covered chamber slide. The cell was treated with IC_50_ concentration of **2**, **3** and **4** lefts for 24 hours incubation. Finally, slide was gently washed with cold DPBS and added 4 % paraformaldehyde for fixation for 60 min at room temperature. Following incubation, the cells were blocked with freshly prepared 3 % H_2_O_2_ in methanol at room temperature. Then, the cells were exposed to permeabilized solution (0.1 % sodium citrate and 0.1 % Triton X-100) for 2 minutes on ice. At this stage in order to prepare a DNase I enzyme treated as positive control, 10 μl of DNase-I and 90 μl its buffer was added to slide and incubated at room temperature for 10 minutes. The compotent cell was transferred into TUNEL reaction mixture (50 μL/section) involving a TdT and fluorescein-dUTP mix for 1 h at 37 °C, in a humidified phase and protected from light. In the end of the process, the positive cell nuclei obtained a green fluorescent signal which visualized by a Leica fluorescent microscope.

**Cell migration assay**

The migration feature of the **2**, **3** and **4** was monitored using the cell migration method. Briefly, an equal number of HeLa cells (3.5 × 10^4^ HeLa cells) were seeded into the two reservoirs separated by a 500 µm thick wall and allowed to grow to 90–95 % confluence. Subsequent to cell growth, the insert was gently removed and treated with IC50 concentrations of **2**, **3** and **4**. The closure of the gap by the cells was photographed 0, 1 and 2 days after incubation by using a phase contrast inverted microscope.

**DNA topoisomerase I enzyme inhibition method**

DNA topoisomerase I enzyme inhibitory activity of **2**, **3** and **4** were observed by using a cell-free topoisomerase I enzyme method kit. DNA topoisomerase I inhibition assay implied to change from super coiled pHOT1 plasmid that is relaxed form in the presence of DNA topoisomerase I enzyme alone and with **2**, **3** and **4**. In brief, 1 µL plasmid pHOT1 DNA in relaxation buffer, 2 U recombinant human topoisomerase I, 2, 3, 4 (at IC50 concentrations), and camptothecin (positive control) were mixed to 20 μL of reaction tube. The reaction was incubated at 37°C for 30 min and then terminated by adding of stop solution with loading dye. After the termination of the process, the samples were analyzed utilizing a 1.5% agarose gel for 50 min. At the end of the electrophoresis assay, DNA bands for this part were stained with ethidium bromide and also photographed with gel imaging system.

**Immunohistochemistry**

Immunohistochemistry (IHC) methods utilized to localize antigens with changing expression level following **2**, **3** and **4** treatments. Indeed, HeLa cells and HT29 (15.000 cells/chamber slide section) conveyed in poly-L-lysine covered chamber slide. The cells were treated with IC50 concentration of **2**, **3** and **4** and remained for 24 h. There was a negative control that had no test compounds. At the end of the incubation time, the chamber was removed from the slide and added 4% paraformaldehyde in DPBS at pH 7.4 for fixation for 60 min at room temperature. Following incubation, heat-induced epitope retrievals (HIER) were performed utilizing visualization and Cell Condition 1 which achieved with the Universal DAB Detection Kit, conforming to manufacturer's instructions. IHC assay was determined using Bcl-2 (clone 124; Ventana, mouse monoclonal), CK7 (clone OV-TL 12/30; Ventana, mouse monoclonal), CK20 (clone Ks20.8; Ventana, mouse monoclonal), and P53 (clone D07; Ventana, mouse monoclonal) on the VENTANA Bench-Mark XT System. The number of positive and negative cells was counted in five zones by repeating three times for each section. The slides were scored staining intensity score rated as follows: weak and moderate staining (1+, <50% positive cells), and strong staining (2+, >50% positive cells). A score of 2+ was considered positive for relevant expression while a score of 1+ was considered negative.

**Preparation of microorganisms and Disc diffusion assay**

In this study, 10 microbial cultures of this part, belonging to ten types of bacterial were used. The culture was grown in Mueller–Hinton Broth (Merck) for all the bacterial strains at 36 °C. Antimicrobial test was carried out by using disc-diffusion assay utilizing 100 µL of suspension with 10^8^ CFU/mL of bacteria and 10^6^ CFU/mL of yeast spread on Nutrient Agar and Potato Dextrose Agar, respectively. Also, the blank disc (Oxoid = 6 mm in diameter) was impregnated with 20 µL of each substance (105 µg/disc) and placed on the inoculated agar. Negative controls (KCN) were prepared using the same solvent (water) employed to dissolve each substance. Cefoperazone (75 µg) (105 µg/disc) + Sulbactam (30 µg) were used as positive references to investigate the sensitivity of a strain of each microbial species tested. The inoculated plate was incubated at 36 °C for clinical bacterial strains. Antimicrobial activities were evaluated by obtaining the zone of inhibitory effect against the test organisms.

**Microdilution assay**

The minimal inhibitory concentration value was studied for the microorganism cells, which evaluated to sensitive the substances and they tested in the disc-diffusion method. Inoculate of microorganism was prepared utilizing 12 h suspensions and broth cultures were adjusted to 0.5 McFarland turbidity standard. Each substance was dissolved in 10% dimethyl sulfoxide which diluted to the highest concentration (1000 µg/mL) for testing. Indeed, serial twofold dilution was made in a concentration range from 3.9–1000 µg/mL in test tubes involving nutrient broth. Also, prepared suspensions for standard microorganisms were added to each dilution in a 1:1 ratio. Growth act of microorganism cells was determined visually after incubation at 36 °C. The minimum concentration was no visible growth which was taken as the MIC.

**DNA/BSA binding and gel electrophoresis studies**

To obtain the interaction act of the molecules with calf thymus DNA and also to determine the binding constants (𝐾𝑏) UV–Visible absorption spectroscopy method was utilized. Indeed, 2.5 mg calf thymus DNA in this part was dissolved in Tris–HCl buffer and stabile pending seven days in the refrigerator. The concentration level of calf thymus DNA was calculated spectrophotometrically assay at 260 nm. This method was done according to previous studies.

**Stability study**

The stability potential of the **1-4** in physiological conditions were obtained through the use of a simple ultraviolet–visible spectrophotometric method. The stock solution of each compound was initially prescribed in 1/9 DMSO/Tris–HCl buffer and then the working solution was prepared by adding 0.1 M phosphate buffer to set the concentrations in the range of 1.95 to 250 µM. As per the test procedure in the 3-day study period, the compounds in physiological conditions were examined with the same intervals for 24 hours.

**Statistical Analysis**

The statistical significances were determined by the one-way method of variance (one-way ANOVA) which followed by Tukey test. Also, data that did not define homogeneity variance were performed by the non-parametric Kruskal-Wallis test. Finally, the SPSS assay for Windows program was used for these analyses.

**Fig. S1.** Structure proposed for complexes **1**−**3**


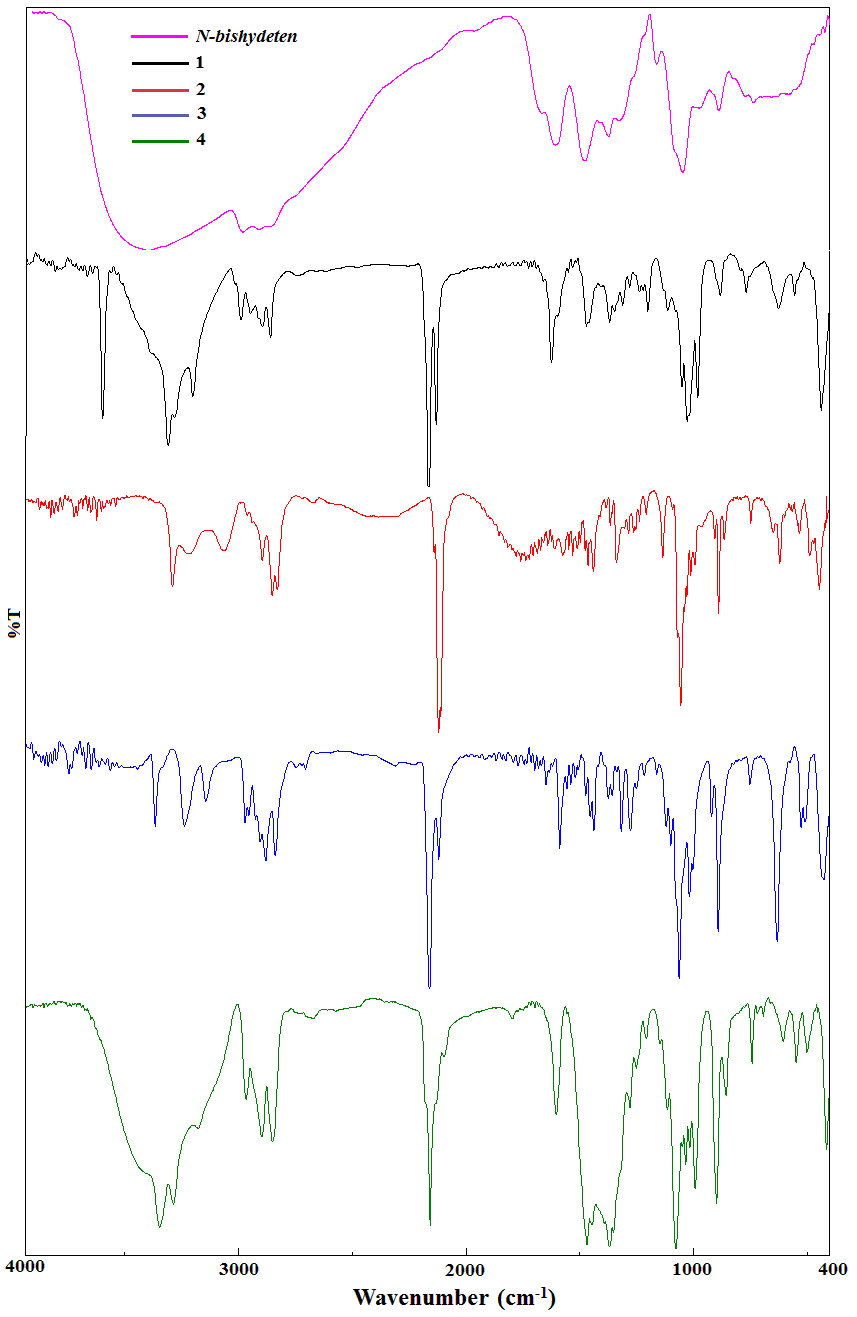


**Fig. S2.** The IR spectra of N-bishydeten ligand and complexes**1−4**


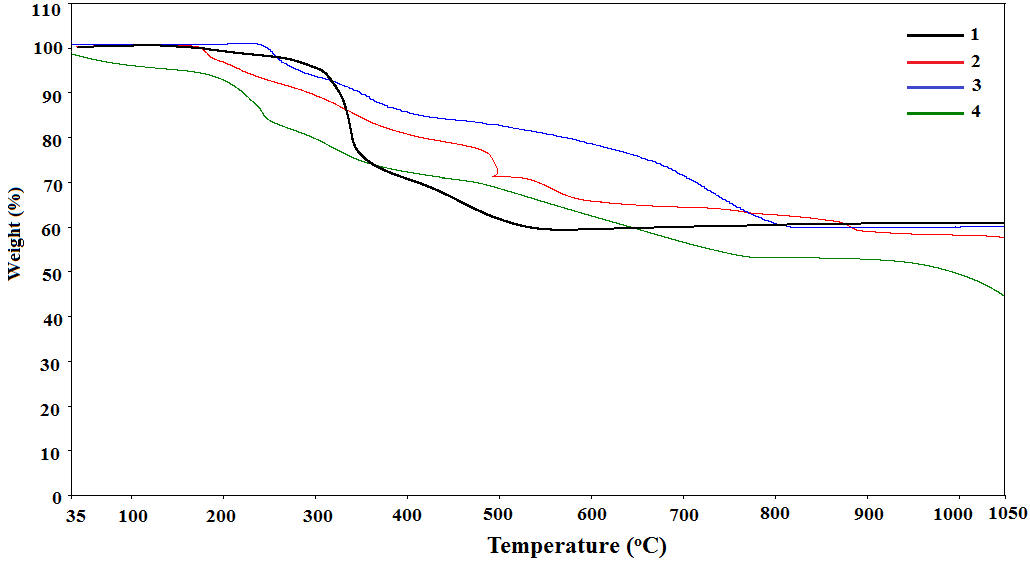


**Fig. S3.** The TG curves of **1**−**4**


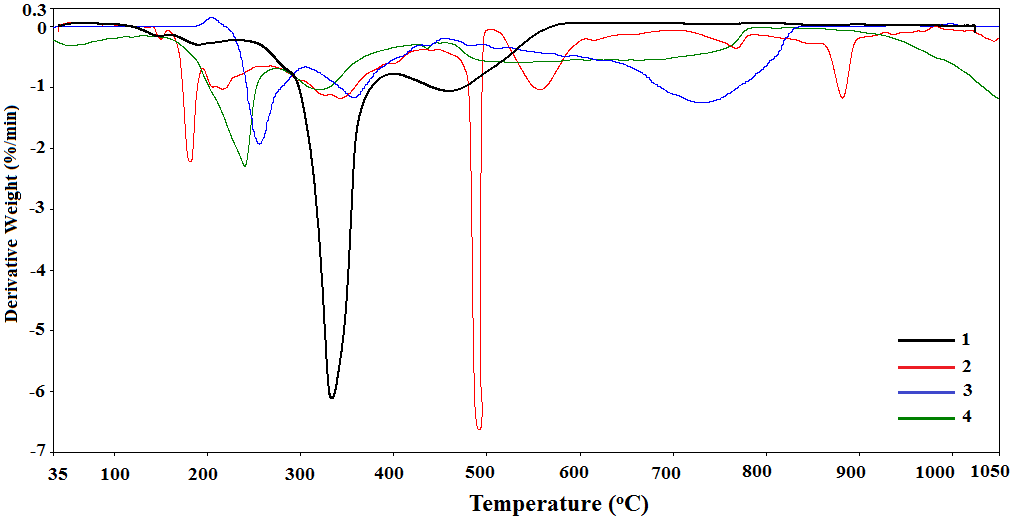


**Fig. S4.** The DTG curves of **1**−**4**


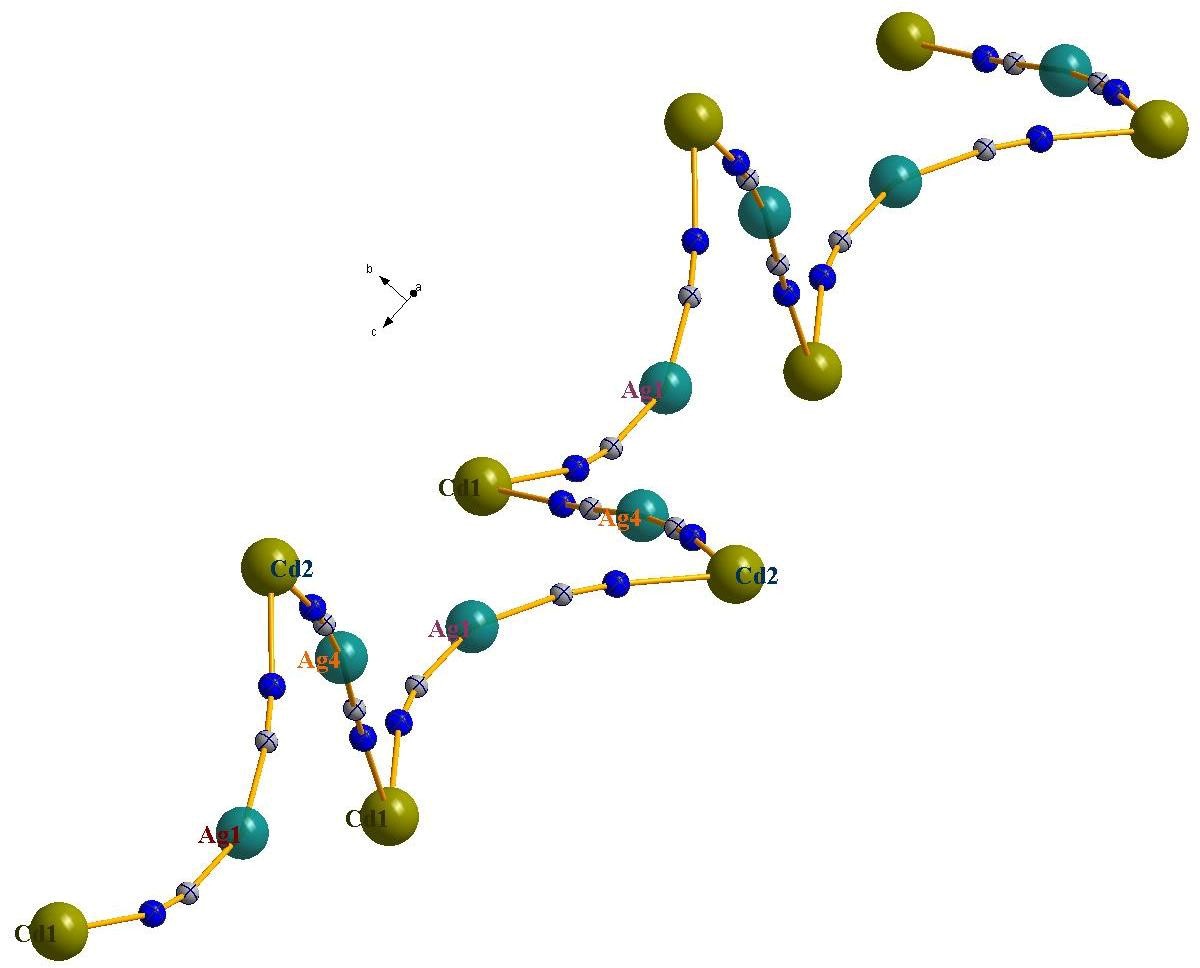


**Fig. S5.** The 3D zigzag chain similar to the "M" shape of complex **4**(The image in figure S5 was generated by using K. Brandenburg, Diamond- Crystal and Molecular Structure Visualization, Crystal Impact GbR, Vers. 4.5.2, Bonn, Germany, 2018.)

**
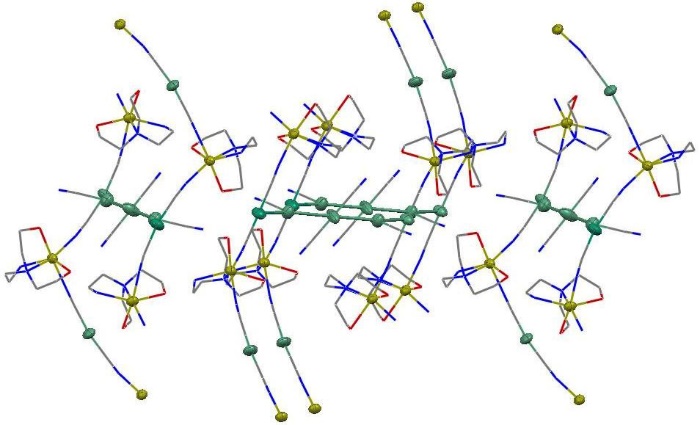
**

**Fig. S6.** The package structure formed by asymmetric units of complex **4**(The image in figure S6 was generated by using K. Brandenburg, Diamond- Crystal and Molecular Structure Visualization, Crystal Impact GbR, Vers. 4.5.2, Bonn, Germany, 2018.)


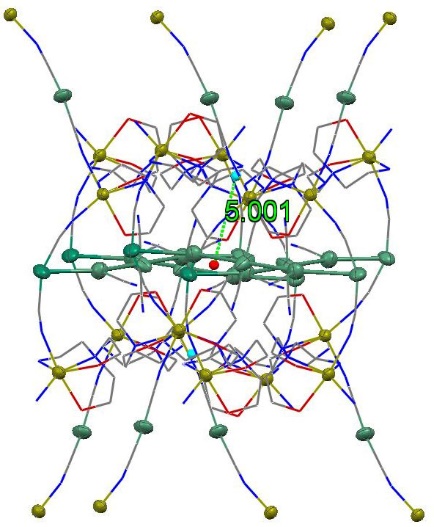


**Fig. S7.** The sandwich-type like structure of complex **4**(The image in figure S7 was generated by using K. Brandenburg, Diamond- Crystal and Molecular Structure Visualization, Crystal Impact GbR, Vers. 4.5.2, Bonn, Germany, 2018.)


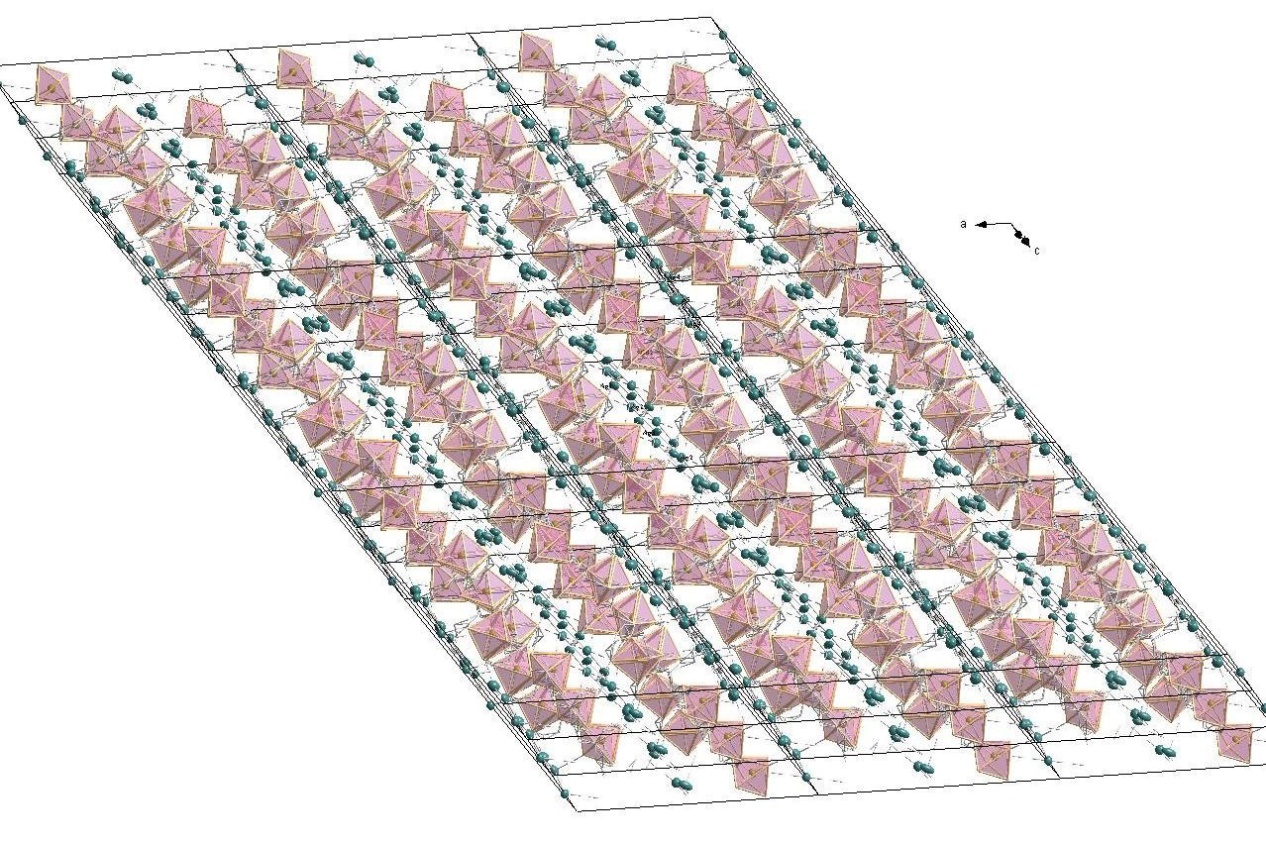


**Fig. S8.** The sandwich-type like 2D structure of complex **4**(The image in figure S8 was generated by using K. Brandenburg, Diamond- Crystal and Molecular Structure Visualization, Crystal Impact GbR, Vers. 4.5.2, Bonn, Germany, 2018.)


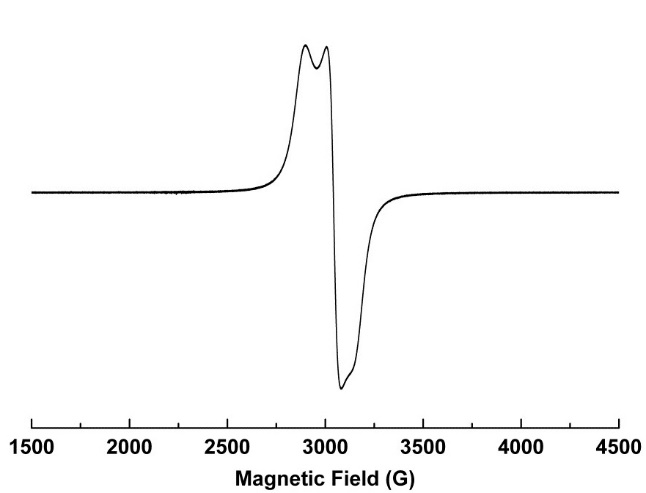


**Fig. S9.** The powder EPR spectrum of complex **2** at room temperature


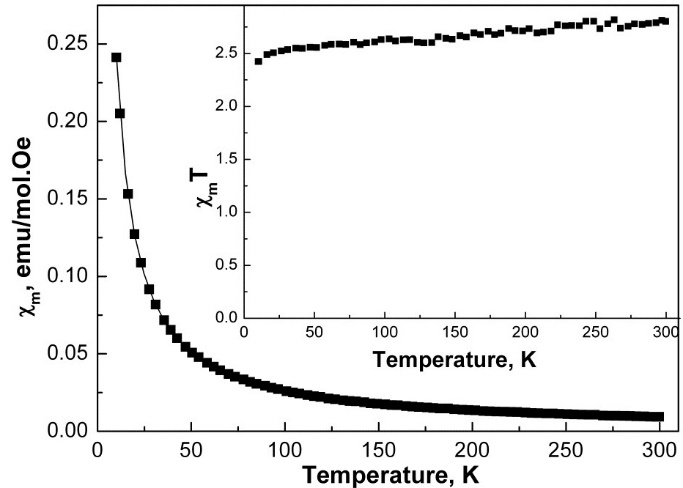


**Fig. S10.** The temperature dependence of the molar magnetic susceptibility χm for complex **1** (solid line represents a fit by the Curie−Weiss law. Inset: The temperature dependence of χmT)


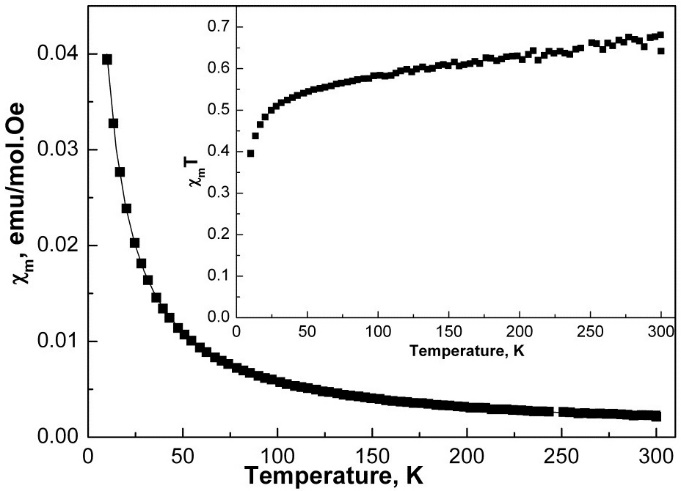


**Fig. S11.** The temperature dependence of the molar magnetic susceptibility **χ_m_** for complex **2** (solid line represents a fit by the Curie−Weiss law. Inset: The temperature dependence of χ_m_T)

**
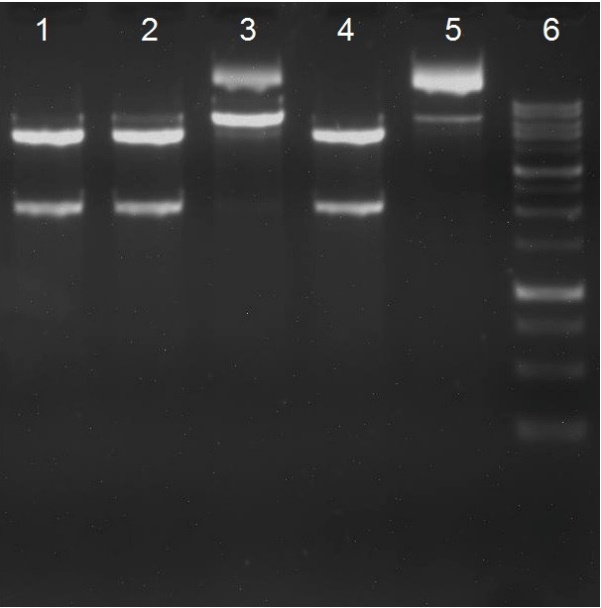
**

**Fig. S12.** Inhibition of *Kpn*I and *Bam*HI restriction endonucleases activity. Following 4 h 37°C digestion of the 14µL with 10U *Kpn*I and *Bam*HI, these digestion products were resolved with 1.5% agarose gel containing ethidium bromide. Lane 1: enzyme + DNA + complex **4**, Lane 2: enzyme + DNA + complex **3**, Lane 3: enzyme + DNA + complex **2**, Lane 4: Positive control (enzyme + DNA), Lane 5: Negative control (plasmid DNA + water); lane 6: DNA marker (1Kb).

**
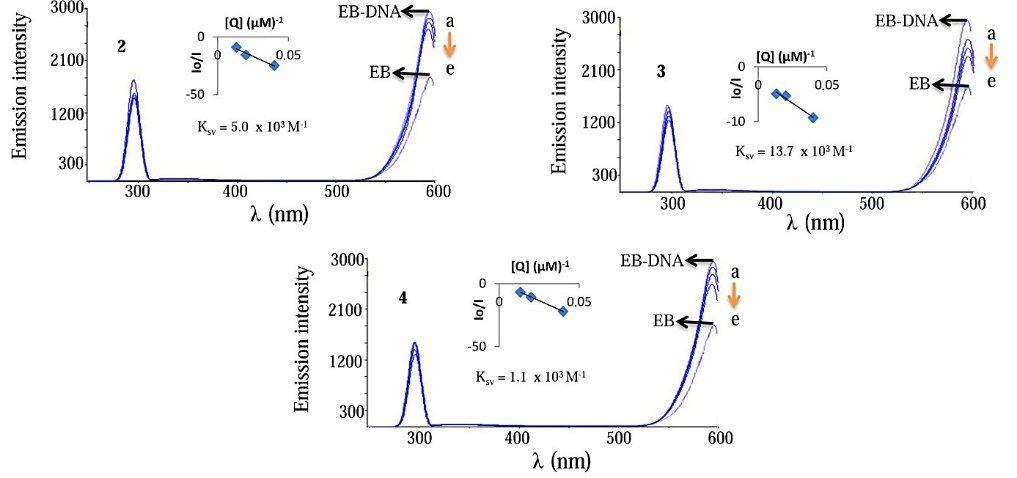
**

**Fig. S13.** The emission spectra of EB-bound (a) DNA solutions in the absence and presence of increasing concentrations of **2**, **3**, and **4** 25 μM (b), 50 μM (c), and 75 μM (d). [EB]=10.0 μM (e), [DNA] 50.0 μM. The arrows show the changes in intensity upon increasing amounts of **2**, **3**, and **4**. Inset shows the plots of emission intensity *I_0_/I* vs. *[Q]* (µM) for determining *K_SV_*.


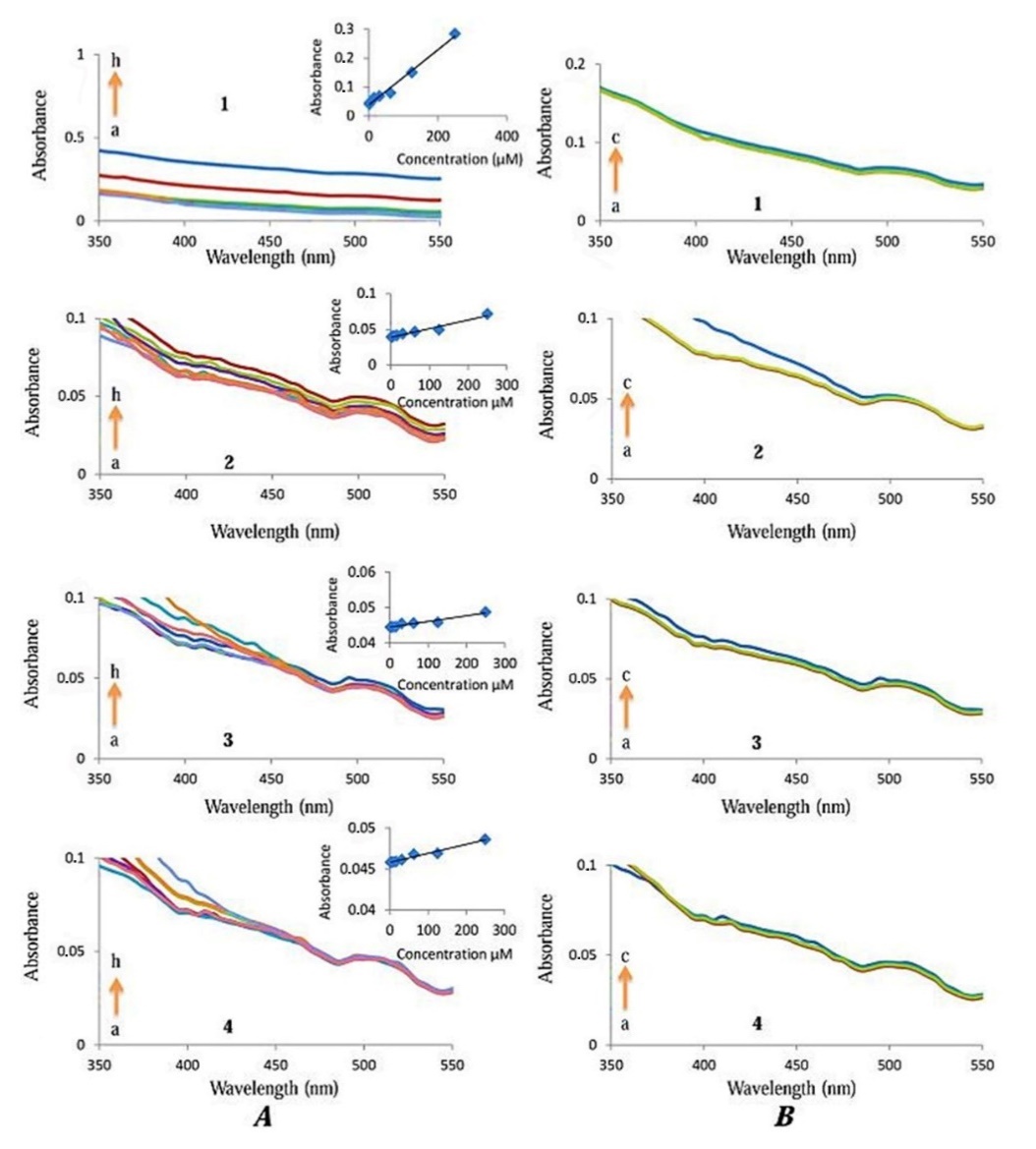


**Fig. S14.** *(A)* The spectrum data of the compounds in following concentrations: 1.95 µM (a), 3.91 µM (b), 7.81 µM (c), 15.63 µM (d), 31.25 µM (e), 62.5 µM (f) and 250 µM (h). *(B)* The stabilities of the compounds (250 µM) in 0.1 M phosphate buffer (pH 7.4) at 37 °C. Spectra collected once every day for day 1 (a), day 2 (b), and day 3 (c).


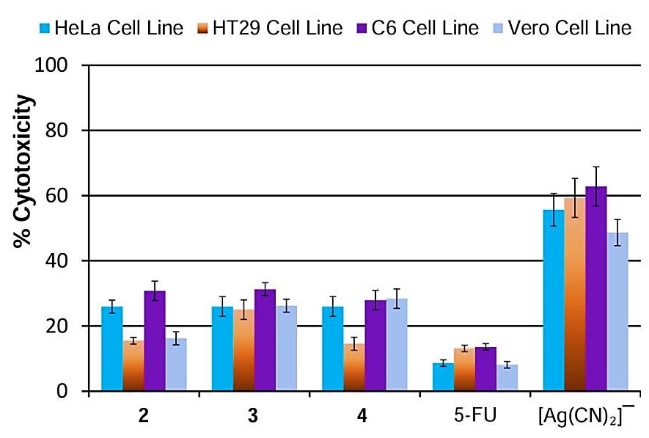


**Fig. S15.** The cytotoxic activity of **2**, **3**, **4** and [Ag(CN)₂]‾ on HT-29, HeLa, C6 and Vero cells. Exponentially growing cells were incubated with IC50 concentrations of **2**, **3**, **4** and [Ag(CN)₂]‾ and cytotoxicity was determined by LDH Cytotoxicity Assay. The % cytotoxicity of **2**, **3**, **4** and 5FU values ranged from 15% to 25%, and a % cytotoxicity of [Ag(CN)₂]‾ values approximately 60%. [Ag(CN)₂]‾ was the most cytotoxic compound (p<0.05) tested against all cell lines. Percent cytotoxicity was reported as mean values ± SDs of three independent assays.

**
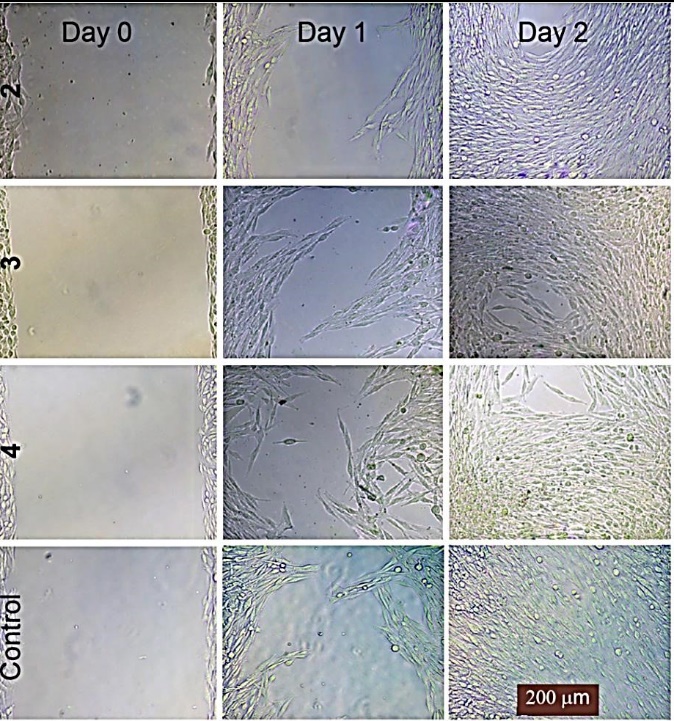
**

**Fig. S16.** Effect of **2**, **3** and **4** on the migration of HeLa cell line. The closure of the HeLa cell line was photographed 0, 1, and 2 days after incubation with Ag(I) compounds at IC_50_ concentrations using a phase contrast microscope (Leica DMIL, Germany) until complete cell closure was observed in the untreated control. Note that anti-migration effect of **2**, **3** and **4** on the cells was more obvious at day 2.


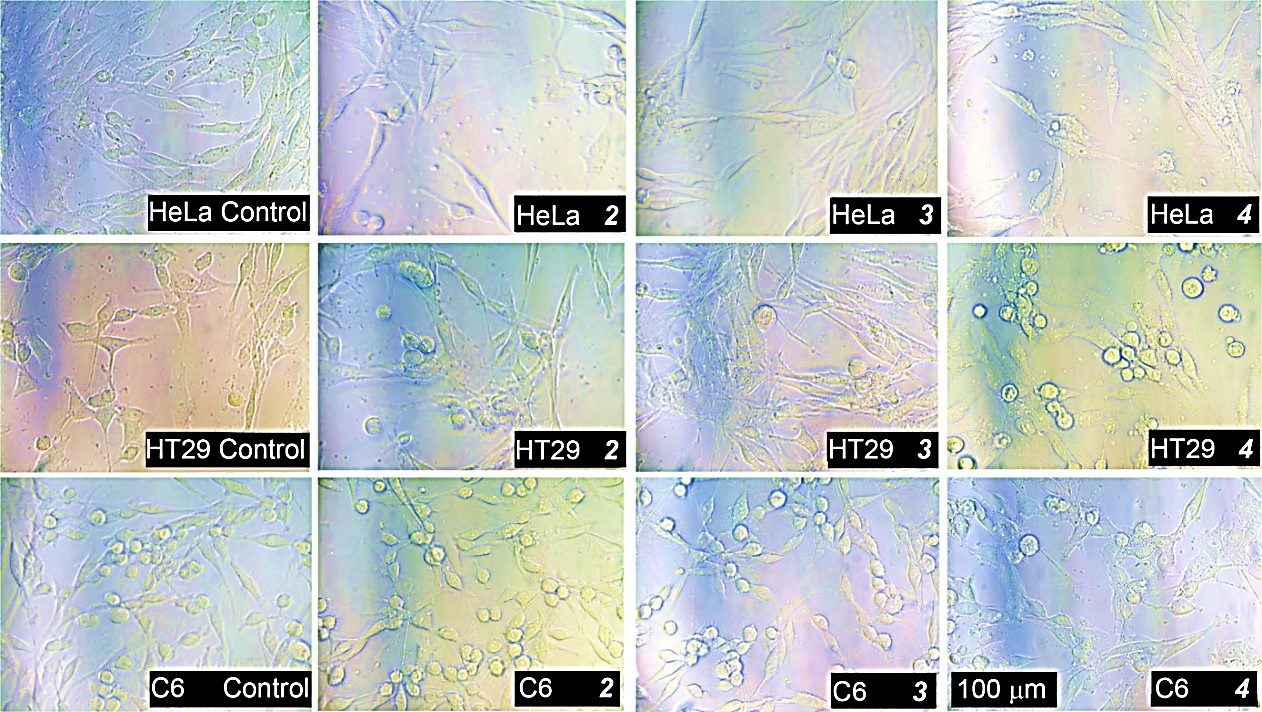


**Fig. S17.** The effect of **2**, **3**, and **4** on the morphology of HeLa, HT29 and C6 cells. Exponentially growing cells were incubated with IC_50_ concentrations of **2**, **3**, and **4** at 37 °C for overnight and visualized by digital camera attached inverted microscope (Leica IL10, Germany). DMSO treated cells as controls. All scales are 100 µm


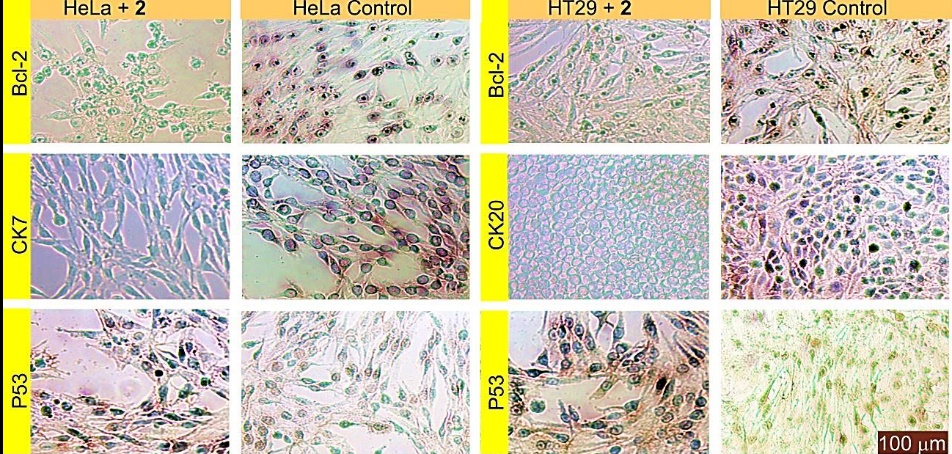


**Fig. S18**. Representative images of the cells treated **2** examined by immunohistochemical staining for functional protein group (Bcl-2 and P53), and for marker protein group (CK7 and CK20). The specific signals are shown as brown staining.


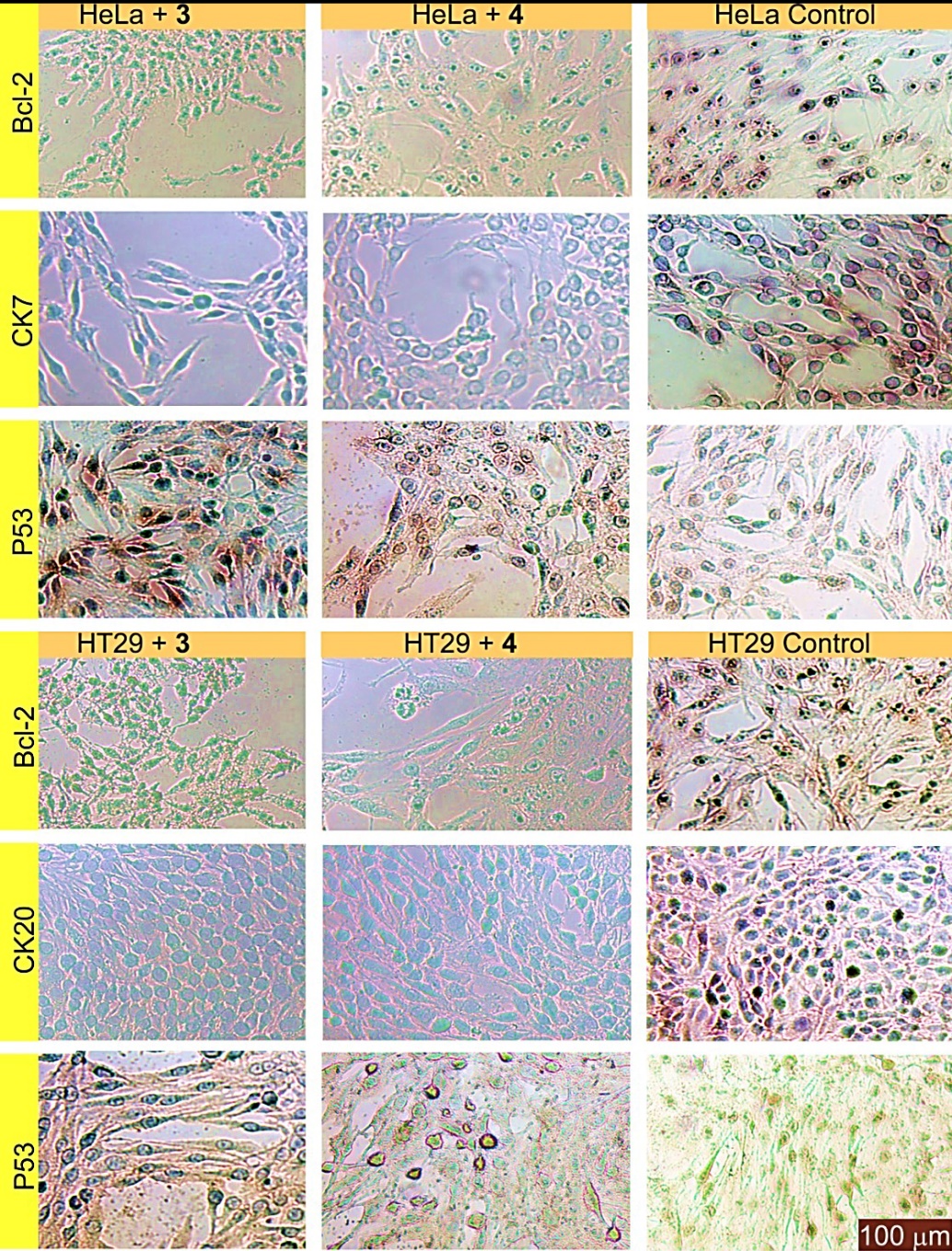


**Fig. S19.** Representative images of the cells treated **3** and **4** examined by immunohistochemical staining for functional protein group (Bcl-2 and P53), and for marker protein group (CK7 and CK20). The specific signals are shown as brown staining. Bar is 100 µm.


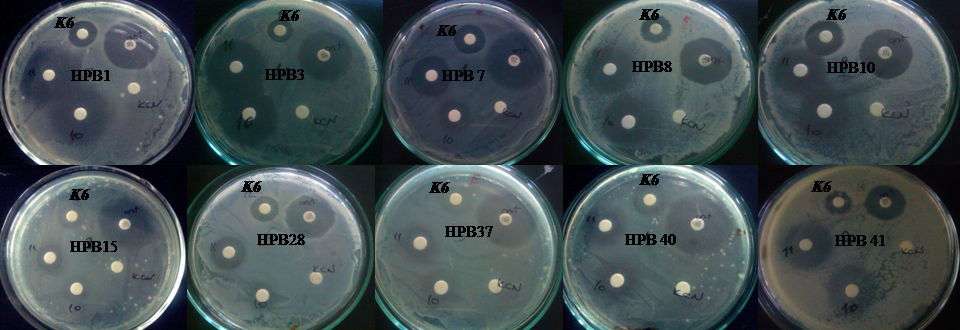


**Fig. S20**. Bacterial inhibition zones of complex ***1*** *(coded as K6)*


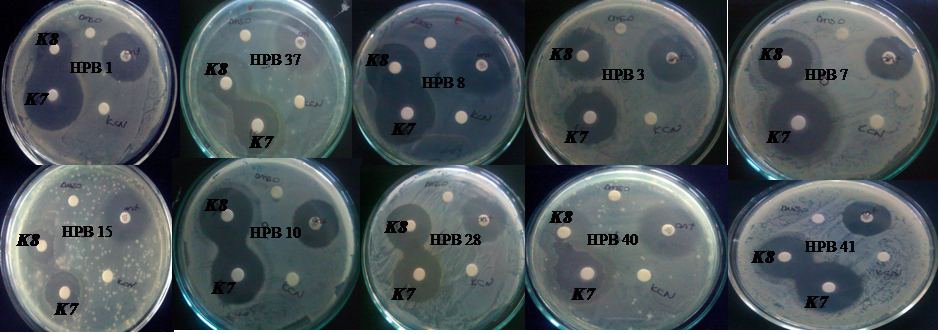


**Fig. S21**. Bacterial inhibition zones of complex ***2*** and ***3*** *(coded as K7 and K8, respectively)*

**
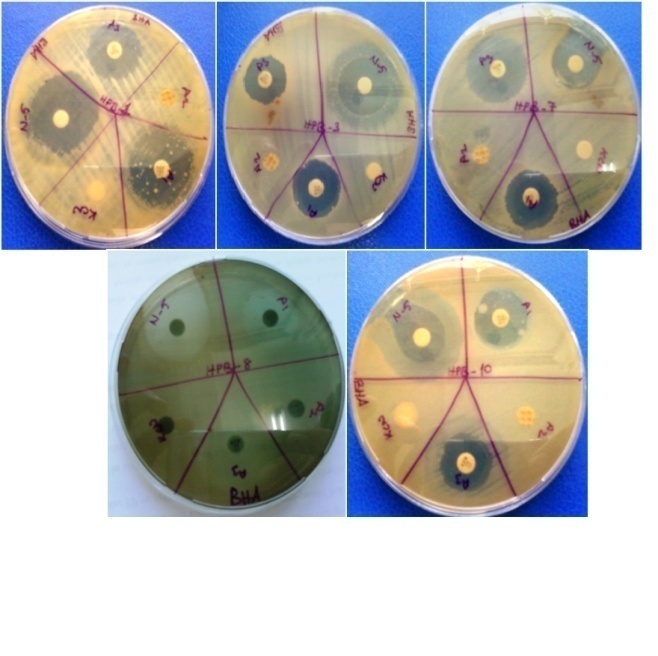
**
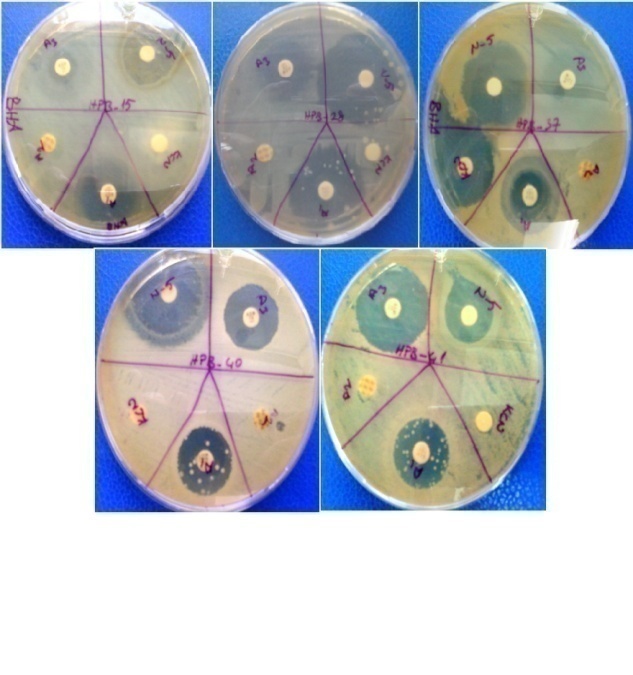


**Fig. S22**. Bacterial inhibition zones of complex ***4*** *(coded as A1)*

**Table S2.** Selected bond lengths (Å) and bond angles (°) of **4**.

| Bond Lengths (Å) | | | |
| --- | --- | --- | --- |
| Cd1-N13 | 2,192(4) | Ag1-C8 | 2.080(5) |
| Cd1-N2B | 2.297(11) | Ag1- Ag2 | 3.1216(7) |
| Cd1-N2A | 2.308(10) | Ag2-C10 | 2.01(3) |
| Cd1-N4 | 2.312(4) | Ag2-C9 | 2.062(7) |
| Cd1-N1 | 2.341(4) | Ag2-N15 | 2.13(2) |
| Cd1-O2A | 2.407(11) | Ag2-Ag3 | 3.2199(5) |
| Cd1-O1A | 2.451(10) | Ag3-C11 | 2.048(6) |
| Cd2-N7 | 2.229(4) | Ag4-C18 | 2.064(5) |
| Cd2-N11 | 2.302(4) | Ag4-C20 | 2.070(5) |
| Cd2-O4A | 2.314(13) | Ag4-Ag5 | 3.2313(5) |
| Cd2-N9A | 2.326(10) | Ag5-C19 | 2.077(6) |
| Cd2-N8 | 2.356(4) | Ag6-N16 | 1.96(3) |
| Cd2-O3A | 2.426(17) | Ag6-C21 | 2.002(8) |
| Ag1-C7 | 2.078(5) | Ag6-C22 | 2.14(3) |
| Bond Angles (^o^) | | | |
| N13-Cd1-N2B | 115.5(7) | Ag1-Ag2-Ag3 | 154.19(2) |
| N13-Cd1-N2A | 116.2(6) | Ag2-Ag3-Ag2 | 180.000 |
| O2A-Cd1-O1A | 90.9(6) | Ag4-Ag5-Ag4 | 180.000 |
| N13-Cd1-N4 | 99.04(17) | C7-Ag1-C8 | 158.4(2) |
| N2B-Cd1-N4 | 86.5(9) | C7-Ag1-Ag2 | 84.45(14) |
| N2A-Cd1-N4 | 86.2(8) | C8-Ag1-Ag2 | 77.48(15) |
| N13-Cd1-N1 | 157.05(18) | C9-Ag2-N15 | 178.0(5) |
| N2B-Cd1-N1 | 77.2(6) | C11-Ag3-C11 | 180.000(3) |
| N2A-Cd1-N1 | 76.5(6) | C18–Ag4–C20 | 171.1(2) |
| N4-Cd1-N1 | 100.84(15) | N11-C18-Ag4 | 177.4(4) |
| N13-Cd1-O2B | 95.7(7) | N12-C19-Ag5 | 175.1(5) |
| N9A-Cd2-N8 | 71.9(4) | N13-C20-Ag4 | 178.6(6) |
| N7-Cd2-N8 | 157.1(18) | N14-C21-Ag6 | 177.4(8) |
| N7-Cd2-O3B | 98.5(7) | Ag1-C8-N7 | 170.4(5) |
| N7-Cd2-N11 | 96.69(16) | Ag4-C20-N13 | 178.6(6) |
| O3B-Cd2-O4A | 90.3(15) | C20-N13-Cd1 | 167.2(5) |
| O4B-Cd2-O3A | 91.9(8) | C18- N11-Cd2 | 171.4(4) |
| N11-Cd2-O4A | 175.6(8) | C8- N7-Cd2 | 170.0(4) |

| **Table S3.** Bacterial strains coded as HPB | | |
| --- | --- | --- |
| HPB-1 | *Staphylococcus aureus* | ATCC 29213 |
| HPB-3 | *Bacillus Subtilis* | ATCC 6633 |
| HPB-8 | *Escherichia coli* | AU Tıp 111 |
| HPB-10 | *Bacillus Cereus* | DSM 4312 |
| HPB-15 | *Enterobacter Aerogenes* | AU Tıp 2924 |
| HPB-28 | *Salmonella Gallinarum* |  |
| HPB-37 | *Pseudomonas Aureginosa* | ATCC 27859 |
| HPB-40 | *Salmonella Enteritidis* | ATCC 13076 |
| HPB-41 | *Steptococcus Pyogerez* | ATCC 176 |
